# Multifunctional nanostructures: Intelligent design to overcome biological barriers

**DOI:** 10.1016/j.mtbio.2023.100672

**Published:** 2023-05-18

**Authors:** Mehdi Azizi, Rana Jahanban-Esfahlan, Hadi Samadian, Masoud Hamidi, Khaled Seidi, Alireza Dolatshahi-Pirouz, Amirhossein Ahmadieh Yazdi, Amin Shavandi, Sophie Laurent, Mahsa Be Omide Hagh, Nahid Kasaiyan, Hélder A. Santos, Mohammad-Ali Shahbazi

**Affiliations:** a Department of Tissue Engineering and Biomaterials, School of Advanced Medical Sciences and Technologies, Hamadan University of Medical Sciences, Hamadan, Iran; b Dental Implants Research Center, Hamadan University of Medical Sciences, Hamadan, Iran; c Department of Medical Biotechnology, School of Advanced Medical Sciences, Tabriz University of Medical Sciences, Tabriz, Iran; d Department of Molecular Medicine, School of Medicine, Hamadan University of Medical Sciences, Hamadan, Iran; e Université Libre de Bruxelles (ULB), École Polytechnique de Bruxelles-BioMatter Unit, Avenue F.D. Roosevelt, 50 - CP 165/61, 1050, Brussels, Belgium; f Department of Health Technology, Technical University of Denmark, 2800 Kgs, Lyngby, Denmark; g General, Organic and Biomedical Chemistry Unit, Faculty of Medicine and Pharmacy, Research Institute for Health Sciences and Technology, University of Mons – UMONS, Mons, Belgium; h Immunology Research Center, Faculty of Medicine, Tabriz University of Medical Sciences, Tabriz, Iran; i Department of Nephrology and Hypertension, University Medical Center Utrecht, 3508 GA, Utrecht, Netherlands; j Department of Biomedical Engineering, University Medical Center Groningen, University of Groningen, Antonius Deusinglaan 1, 9713 AV, Groningen, Netherlands; k W.J. Kolff Institute for Biomedical Engineering and Materials Science, University of Groningen, University Medical Center Groningen, Antonius Deusinglaan 1, 9713 AV, Groningen, Netherlands; l Drug Research Program, Division of Pharmaceutical Chemistry and Technology, Faculty of Pharmacy, University of Helsinki, FI-00014, Helsinki, Finland

**Keywords:** Nanoparticles engineering, Cancer, Nanotechnology, Targeted drug delivery, Biological barriers, Biotechnology

## Abstract

Over the past three decades, nanoscience has offered a unique solution for reducing the systemic toxicity of chemotherapy drugs and for increasing drug therapeutic efficiency. However, the poor accumulation and pharmacokinetics of nanoparticles are some of the key reasons for their slow translation into the clinic. The is intimately linked to the non-biological nature of nanoparticles and the aberrant features of solid cancer, which together significantly compromise nanoparticle delivery. New findings on the unique properties of tumors and their interactions with nanoparticles and the human body suggest that, contrary to what was long-believed, tumor features may be more mirage than miracle, as the enhanced permeability and retention based efficacy is estimated to be as low as 1%. In this review, we highlight the current barriers and available solutions to pave the way for approved nanoformulations. Furthermore, we aim to discuss the main solutions to solve inefficient drug delivery with the use of nanobioengineering of nanocarriers and the tumor environment. Finally, we will discuss the suggested strategies to overcome two or more biological barriers with one nanocarrier. The variety of design formats, applications and implications of each of these methods will also be evaluated.

## Graphical abstract

TOC: Despite cutting-edge original high-quality investigations on targeted drug delivery, there is still limited attention to the significant role of technologies in bypassing or alternating the biological barriers for the success of cancer therapy. This review focus on the characteristics of biological barriers and chemical aspects of manipulating drug delivery carriers to overcome these barriers.

**Figure:**
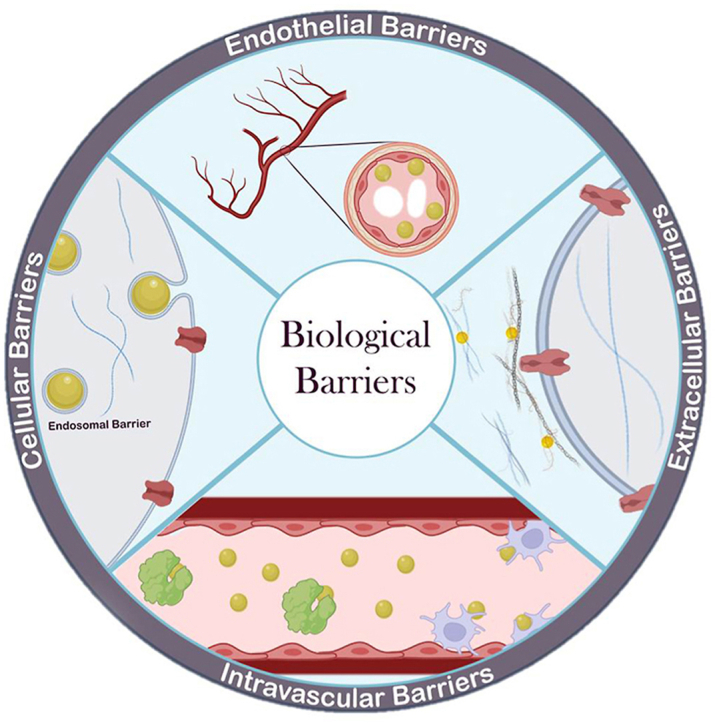

## 1 Introduction

Solid tumors are analogous to aberrant organs in that blood vessels nourish them and lymphatic vessels drain them, and in that the cancer cells as well as various other host cells are embedded in an extracellular matrix (ECM) in their microenvironment [1]. Therefore, therapeutic agents are required to cross both the vessel wall and the ECM when traveling from circulation to the cancer microenvironment [2]. Confined spaces, in which tumors grow, as well as deposited collagen and hyaluronan as matrix components, all together induce solid stressors, compressing blood and lymphatic vessels and impairing their function [3]. As tumor vessels are leaky, tumor blood flow is impaired, thus increasing intratumoral fluid pressure. Such unusual blood flow compromise the drug delivery and facilitate hypoxia, the latter of which itself leads to the invasion and metastasis of tumors, immunosuppression and treatment resistance, inflammation, and fibrosis [4]. Some of the FDA-approved cancer nanotherapeutics, which take advantage of the enhanced permeation and retention (EPR), accumulate passively around leaky tumor vessels. The unimpressive results may be attributed to the physiological barriers hindering nanotherapeutic delivery to the tumor site [5]. Part of this is related to the stiffness of the ECM, which in fact paves the way for the emergence of desmoplastic tumors [6]. For instance, collagen, hyaluronan, and some other factors have been observed to accumulate in a parallel manner in pancreatic tumors [7]. However, a dense ECM is not thought to be the only delivery barrier in the tumor microenvironment (TME), since abnormal vasculature, abundant stroma cells, heterogeneous blood flow, and high interstitial fluid pressure have been demonstrated to impair the effective extravasation and vascular and interstitial transport of nanomedicines into the TME [8]. In addition, the physiochemical characteristics (e.g., size, surface chemistry, and shape) of the nanocarrier (NC) should be fine-tuned to maintain their stability while enabling them to overcome such barriers [9,10].

Formulations based on nanocarriers have turned into a leading delivery approach for cancer therapy and imaging. Whether these formulations can be successful depends on their innate capabilities, such as utilizing passive targeting by the EPR effect or active targeting by various tumor-specific receptor coatings on the surface of the NC [11,12]. Unfortunately, such methods often do not prove successful in providing a uniform distribution of the NC within the tumor, resulting in insufficient drug concentration locally. Indeed, heterogeneous drug distribution is among the main reasons that nanodrug delivery platforms fail in the clinic [13].

Some of the studies discussed the interaction of biological barriers and nanocarriers. To address this, Van der meel et al. propose that four strategic pillars involving patient stratification, rational drug selection, combination therapies, and immunotherapy can if used properly improve the translation and exploitation of nanomedicine [14]. In another study, Anchordoquy and coworkers focused on the biological, toxicological, immunological, and translational aspects of nanomedicine [15]. Mitchell et al. reviewed the biological barriers to precision medicine applications, emphasizing FDA-approved NPs [16]. There is no in-depth discussion of the role of tumor heterogeneity indices. This review aims to be a comprehensive, authoritative, critical, and easy-to-read review for a broad audience of tumor biologists, material scientists, clinicians, and artificial intelligence experts. First, we present effective strategies of nanobiotechnology-based approached for increasing the NP half-life in the bloodstream and its penetration into cells and tissues (with a focus on the approach to overcome the biological barriers). Second, we highlight the role of intra/inter-tumor (ecological and evolutionary) heterogeneity indices in the efficacy of tumor-localized nanomedicines. Finally, we propose that understanding the interaction between tumor heterogeneity and NSs can provide a roadmap for personal medicine and clinical translation of targeted nano-drugs.

In this review, we highlight the current barriers and available solutions as a way to pave the way for approved nanoformulations. Furthermore, we aim to discuss the main solutions to solve inefficient drug delivery with the use of nanobioengineering of nanocarriers and the tumor environment. Finally, we will discuss the suggested strategies to overcome two or more biological barriers with one NC. The variety of design formats, applications and implications of each of these methods will also be evaluated.

## 2 Challenges associated with the nanoparticles journey in body

**Fig. 1:**
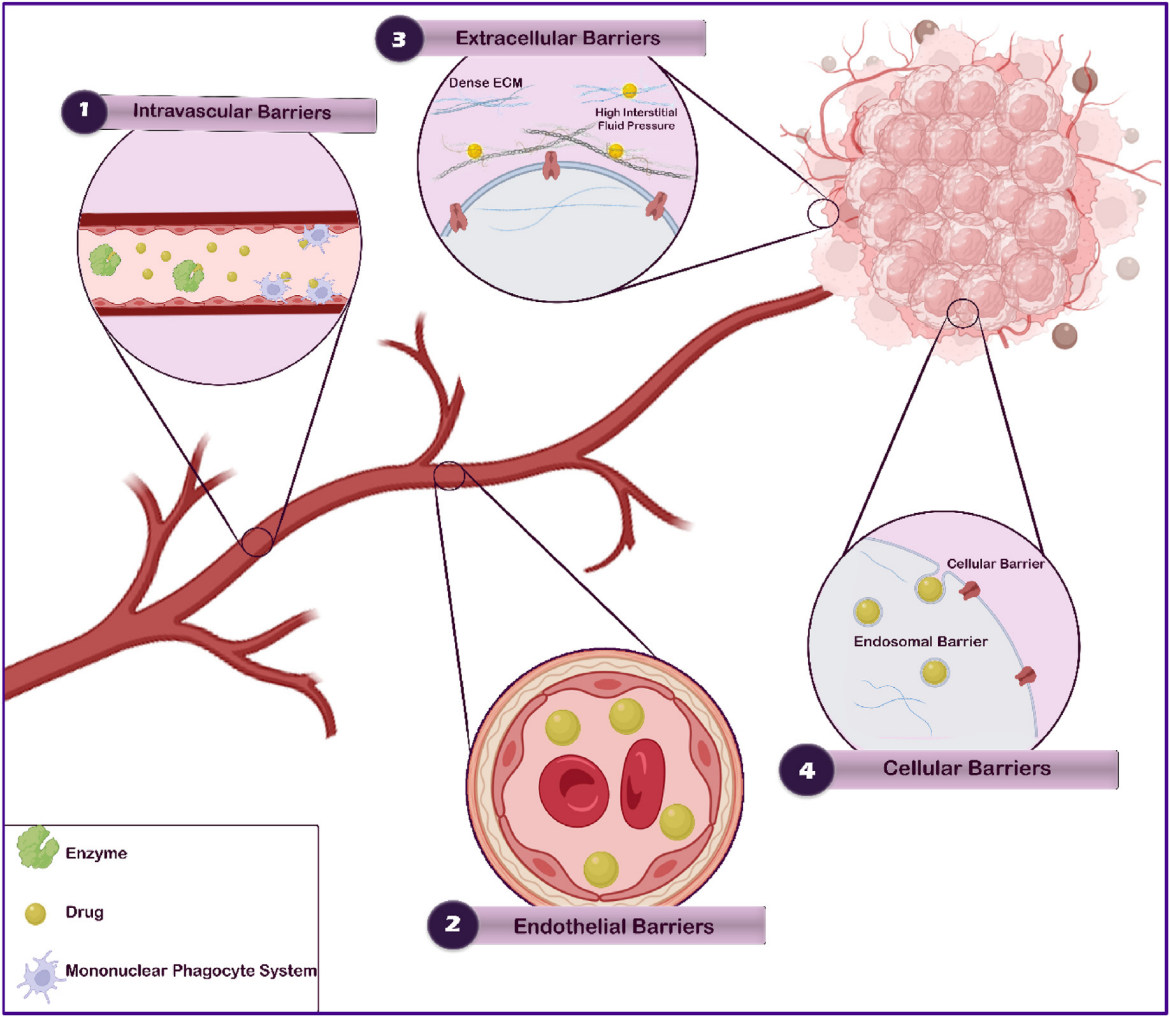
Four major levels of biological barriers. (1) intravascular barriers, (2) endothelial barriers, (3) extracellular barriers, and (4) cellular barriers. Depending on the depth of the target region, the number of biological barriers differs. Organs are easy sites to deliver nanodrugs, while subcellular organelles are difficult because nanodrugs must cross more barriers to reach the targeted region.

Systemically injected NPs must cross several biological/pathophysiological barriers to reach the target region in the tumor site. In this section, we classify these barriers into four main categories, including (for more detail see Ref. [17]): (i) intravascular barriers, (ii) endothelial barriers, (iii) extracellular barriers, and (iv) cellular barriers (Fig. 1). Successful crossing of each of these barriers will be determined by (i) NP half-life in circulation, (ii) specific homing and accumulation, (iii) diffusion and deep penetration, and (iv) uptake and internalization into cancer cells (Table 1).

**Table 1:** Some multifunctional nanoparticles to overcoming of biological barriers.

Type of barriers	Nanostructure	Function	Ref.
Intravascular barriers	Choline-based zwitterionic copolymer-coated ZIF-8	Prolonged blood circulation	[18]
	Soft single molecule polymer nanomaterials	Prolonged blood circulation	[19]
	Silica-based rebuilt RBCs (RRBCs)	Prolonged blood circulation, specific recognition	[20]
	RBC-hitchhiking (RH)	Prolonged blood circulation	[21]
	DOX-PLGA NP	Prolonged blood circulation, immune escape specific recognition	[22]
	Cell membrane-camouflaged nanoparticle	Long circulating NCs and targeted delivery	[23]
	Macrophage plasma membrane-coated albumin nanoparticles	Prolonged blood circulation and selective site tumor accumulation	[24]
	HepM-TSL biomimetic NCs	Prolonged blood, targeted drug delivery	[25]
	NC@anti-CD63	The protein corona shield, targeting abilities	[26]
	Clodronate liposomes prior to administration of AuNPs	Deplete splenic and liver macrophages	[27]
	Ni-liposomes and CREKA-SPIO NPs	5-fold increase in the half-life	[28]
	WT- RBC-derived nanovesicles Clodronate-loaded CD47	Camouflage ‘don't eat me’ signal, internalized by macrophages	[29]
Nanoparticles localization	Fluorescent dye-labeled glycol chitosan NPs	Enhances NP penetration into the tumor core and decreasing tissue stiffness	[30]
	Microbubbles@ DOX- HER2-positive	Disrupt blood–brain/blood–tumor barriers	[31]
	Dox-loaded hybrid micelles with copper ions-RGD	Prolonged blood circulation half-life, bind to integrin receptors	[32]
	Poly (l-glutamic acid)-CA4	Enhance CA4 accumulation and retention in tumor tissue	[33]
	PCL/PLGA NPs	‘Multistage systems’, which allow for the sequential release of different drugs	[34]
	CA4P and docetaxel (DTX)-loaded microspheres	Vascular disruption and cut off the tumor nutrient pathway connected to other tissues	[35]
	Fibrinogen-conjugated AuNPs	Rapid NP accumulation, promote photothermal ablation	[36]
	CREKA-coated iron oxide NPs and liposomes	Home to tumor vessels, a strong inhibitor of clotting	[28]
	Gold nanorods- tTF-RGD	Homing to tumors, induce in situ clot formation	[37]
	DOX and thrombin-loaded chitosan nanoparticles	Home NPs to fibrin–fibronectin complexes found abundantly in the tumor stroma and tumor vessel walls.	[38]
	Polymer-modified magnesium silicide (Mg_2_Si) nanoparticles	Used as deoxygenation agents and as materials that form anti-capillary agents	[39]
	MSCs loaded with chlorin e6-conjugated polydopamine NPs (MSC-PDA-Ce6)	Accumulate and deeply penetrate the lung tumors of mice	[40]
	Paclitaxel (PTX)-encapsulating pH-sensitive micelles	Homing to primary tumors	[41]
Nanoparticles penetration	Collagozome and PTX micelles	Increase uptake of PTX and tumor suppression	[42]
	Au@silica nanorods coated with triphenylphosphonium bromide loaded with thermosensitive S-nitrosothiols	Break collagen fibers and enhances cellular internalization by relaxing stiff ECM	[43]
	Losartan and PTX- pH-sensitive cleavable liposomes	Losartan improved oxygen distribution in tumor tissues and allowed for deep percolation	[44]
	PH20 (rHuPH20) on the surface of PLGA-PEG NPs	Increased the serum half-life of rHuPH20 and quadrupled the accumulation and uniform tumor distribution	[45]
	PEGylated polyethylenimine (PP)-coated NPs of gold/siRNA	Inducer of the quiescence of PSCs and enhanced adsorptive endocytosis	[46]
	Fe_3_O_4_ NPs- anti-epithelial cell adhesion molecule antibody@ DOX-laden multi-walled carbon nanotubes (CNTs)	Self-oxygen bubble generation ability from tumor-enriched H_2_O_2_ for deep penetration	[47]
	Size-changeable graphene quantum dot (GQD) nano-aircraft (SCNA)	Penetrate tumors and deliver anticancer drugs	[48]
	DOX@MSN-amide-WS2-HP	Target deep-seated tumor	[49]
Intracellular Barriers	PLGA-PEG-ITEM4	Tumor cell–specific uptake, an improved blood circulation time, and possible biodistribution	[50]
	Multivalent magnetic NPs coated with varied numbers of the multivalent dendritic polyethyleneimine ligand	Induce steric hindrance in the confined binding area	[51]
	Exosome-coated DOX@E-PSiNPs	Which increase blood vessel extravasation, tumor accumulation, deep tumor penetration, and cellular absorption	[52]
	RGD-coated polymer-lipid hybrid NPs loaded with DOX and mitomycin C (RGD-DMPLN)	Enhance nanodrug accumulation and penetration	[53]

### 2.1 Engineering of the long-circulating nanoparticles: crossing the intravascular barriers

The first barrier to be overcome by systemically administered NPs is to avoid opsonization (adsorption of complement proteins and immunoglobulins act as opsonins) and phagocytosis and being cleared by the reticuloendothelial system (RES). Equally, nonspecific adsorption of plasma proteins forming a “protein corona” is another main barrier that not only affects NP distribution but also negatively impacts targeted drug delivery by concealing tumor-specific targeting moieties. The use of shielding moieties such as PEGylation, plasma protein-derived NPs (e.g., albumin NPs), and tuning nanoparticle physiochemical features (shape, charge, size, and elasticity), as well as the use of self-peptides derived from live cells/organisms or their biomimetics are viable options to prolong NP circulation time [54]. In an advanced step, promoting predefined NP corona formation can be used to fine tune NP behavior and interactions in biological systems [55].

#### 2.1.1 Engineering of NPsʼ physicochemical properties

Modification with compounds like polyethylene glycol (PEG), hyaluronic acid (HA) or polyvinyl pyrrolidone (PVP) is a common way to promote the steric effect and hydrophilicity of NP surfaces. This promotes stealth properties to resist protein adsorption. Currently used as a gold standard to reduce off-target uptake and distribution of NPs, studies show the inadequacy of PEG modifications to achieve anti-biofouling effects. In addition, PEG can trigger activation of the complement system, which results in NP opsonization [56]. Thus, extra modifications are necessary in addition to NP PEGylation. For one, the conjugation of a second PEG layer on conventional PEGylated NPs at a low yet close density to the mushroom-to-brush transition regime produces a dense and dynamic shield on the NP surface and prolongs the NP half-life by reducing their uptake by non-Kupffer liver sinusoidal endothelial cells (LSECs) [57]. Additionally, to prolong the NP half-life, the formation of a dense brush-like shell on the surface of small magnetic NPs has been reported using PEG-neridronate [58]. Alternative polymers like polyglycerol (PG) grafting have shown better performance toward PEG to completely avoid protein corona formation and evade macrophage uptake [18]. Another strategy exploits protein corona formation to promote the stealth effect of PEG and polyphosphoester (PEEP). Preadsorption of clusterin (apolipoprotein J) can reduce nonspecific cellular uptake of polymeric NCs [19]. Similarly, NP PEGylation along with coating the NP surface with ‘don't-eat-me’ signals (CD47 proteins) can preferentially reduce M1 macrophage phagocytic activity [59].

Zwitterionic polymers exhibit prolonged blood circulation as they are highly hydrated due to strong electrostatic interactions between equal cationic and anionic groups. Among these, a phosphoryl choline-based zwitterionic copolymer-coated ZIF-8 nanodrug and DOX have been prepared that exhibit outstanding prolonged blood circulation 48 ​h post-injection [60]. Additionally, cell membrane-inspired chemistry has been attempted using 2-methacryloyloxyethyl phosphoryl choline (MPC) polymers. Such bioinspired polymers can promote minimum contact with blood proteins and resist surface-induced clot formation in the absence of an anticoagulant [61]. Interestingly, a new class of dendrimer-like, highly hydrophilic (enriched with OH- groups) soft single molecule polymer nanomaterials (SMPNs) have been reported to elicit ultra-long circulation (t_1/2β_ ∼65 ​h for SMPN-1) without the need for additional modifications. This suggests their inherent ability to minimize opsonization and evade immune-mediated clearance. Proteomic studies reveal that this unique property stems from continuous remodeling of protein opsonins, including immunoglobulins, complement, and coagulation proteins, and is dominantly ruled by the molecular weight of the SMPNs rather than a similar surface chemistry [62]. In the case of NP geometry, compared to spherical shapes, ellipsoidal, rod-like and tubular-shaped morphologies show better repulsion in circulation. With this in mind, changing the shape of the polymerosome, made up of amphiphilic block copolymers, into ellipsoidal or tubular-shaped morphologies has been reported by utilization of aromatic interactions. This was achieved by anisotropic membranes by exploiting hydrophobic directional aromatic interactions between perylene polymer units within the membrane structure [63]. Acting as a Trojan horse, the use of whole live cells is another promising strategy to improve the NP half-life irrespective of its shape [24].

#### 2.1.2 Bionic strategies

**Fig. 2:**
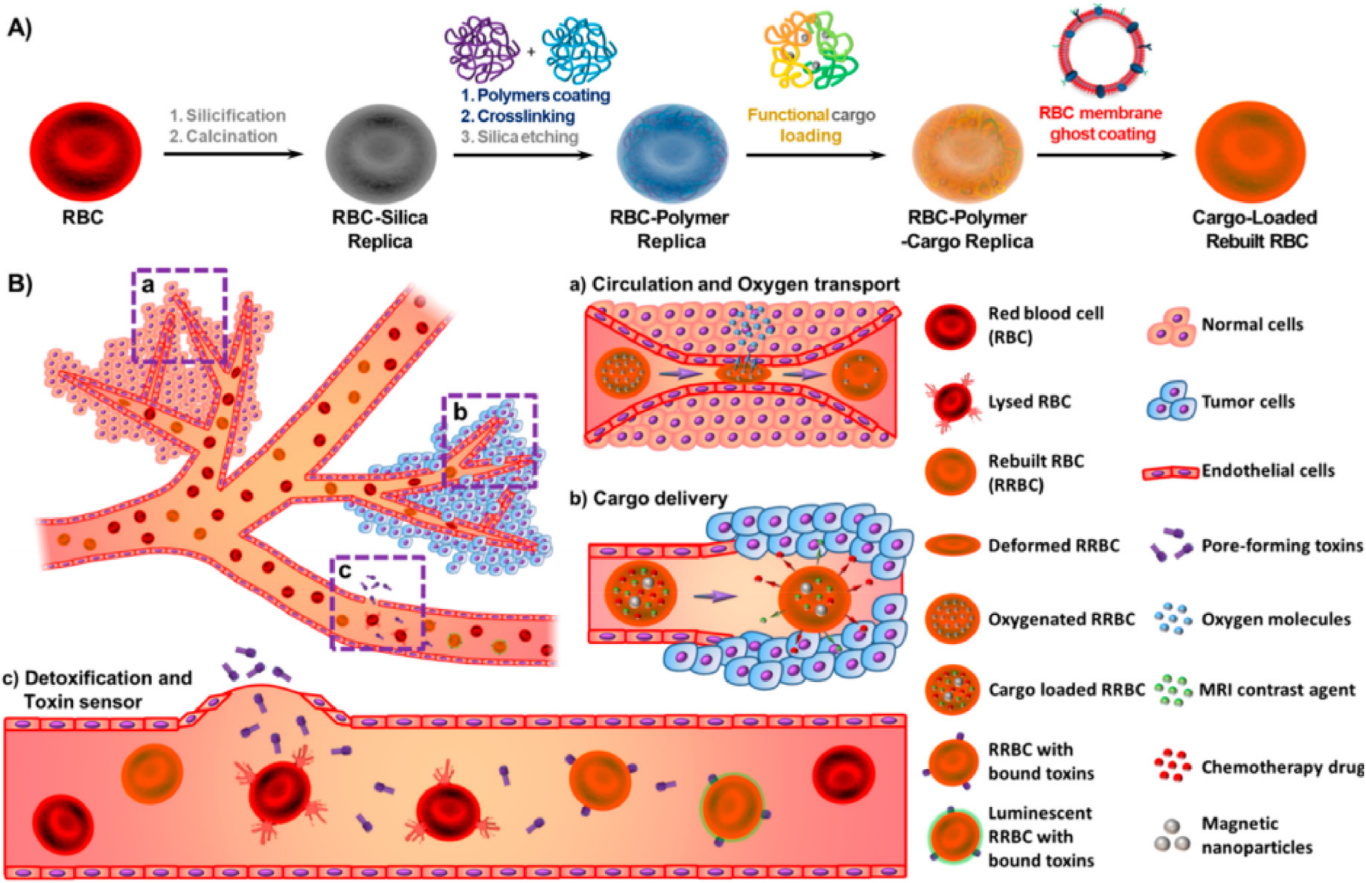
**Artificial RBCs enable longer circulation of NPs.** (A) Construction and properties of resultant RRBC, (B) (a–c), Circulation, oxygen delivery potential and other biomedical application. Reprinted with permission [23]. Copyright 2020, American Chemical Society.

As a result of their membrane structure and surface antigens, active cell membrane–cloaked NPs possess superior properties, such as immune escape, prolonged blood circulation, and specific recognition [64]. Various cells are utilized in this way, including red blood cells (RBCs), platelets [65], immune cells [20], cancer cells [21] and stem cells [22]. Given their unique inherent properties, NP integration with these bodily friendly tools can result in NCs with long circulation times. For instance, integration of the self-recognition marker CD47 in silica-based rebuilt RBCs (RRBCs) prevents phagocytosis by macrophages, leading to a long half-life of 41.8 ​h in vivo (Fig. 2) [23].

In another interesting formulation, a RBC is used to carry NCs on its surface, termed “RBC-hitchhiking (RH)”, in which NCs adsorbed onto the RBCs transfer from RBCs to the first organ downstream of the intravascular injection [25]. Likewise, an erythrocyte hitchhiking DOX-PLGA NP assembly is used to combat melanoma lung metastasis at early and later stages. This RH system not only prolonged the NP half-life but also resulted in 10-fold higher drug percolation in the lung compared to the free NPs [66]. Another formulation called ‘cell membrane-camouflaged nanoparticle’ extracts the cell membrane of live cells to be used as long circulating NCs for NP/drug delivery. Due to the membrane structure and membrane antigens, such an active system can achieve special properties, such as prolonged blood circulation, immune escape and specific recognition [67]. Likewise, macrophage plasma membrane-coated albumin nanoparticles are used to achieve prolonged blood circulation and selective site tumor accumulation targeted drug delivery for the treatment of malignant melanoma in mice [20]. HepM-TSL biomimetic NCs composed of thermosensitive liposome (TSL) vesicles loaded with DOX and ICG and coated with homotypic cancer cell membranes are used for synergistic chemo-photothermal therapy of recurrent hepatocellular carcinoma (HCC) [68]. Additionally, gelatin nanogels shielded with mesenchymal stem cell (MSC) membranes have been developed. Such NCs display a vast pool of molecular recognition moieties, can preserve MSC tumoritropic properties, and can effectively avoid RES clearance to deliver drug-loaded nanogels to the tumor site [69].

#### 2.1.3 Engineering and customizing the protein corona shield (PCS)

Protein corona formation, if properly understood in order to be adopted in the design of NP surface chemistry, is a recently appreciated tactic. Corona formation to favor nanodrug delivery requires prior knowledge to tailor the composition and function of the protein corona, as well as the cellular recognition of nanomaterials to modulate NP interactions. This can be predicted using machine learning methods or obtained by experimental studies [70]. For one, precoating nanoparticles with, for example, plasma immunoglobulin (IgG), forms a stable protein corona, and reintroduction into the plasma can effectively evade immune cell recognition [26]. Similarly, human plasma protein-derived artificial coronas can drastically improve the blood residency and circulation lifetime of liposomes by reducing their interaction with and sequestration by circulatory leukocytes in vivo [71].

Preadsorption of proteins that form biomolecular coronas on NP surfaces can benefit from the intrinsic/active targeting abilities of adsorbed proteins/antibodies. PC-mediated targeted therapy can be achieved on the condition that the proper orientation and accessibility of proteins is preserved to enable correct NP directing to target cells [55]. Along this line, attempts for desired cell targeting by precoating silica NPs with gamma-globulins to form predefined opsonin coronas on the NP surface have failed, since other plasma-adsorbed proteins counteracted the binding of NP-adsorbed immunoglobulins to macrophage surface receptors, and thus, hindered opsonin-induced by macrophages [72]. Another strategy to reduce the shielding effect on NP mistargeting exploits a non-coupling method to decorate NC surfaces with targeting antibodies (anti-CD63) without affecting their functionality, which occurs during the bioconjugation/coupling process or because of protein corona formation. Interestingly, in the presence of 100% serum/plasma, NPs synthetized by chemical engineering lose their targeting abilities, while CD63 antibodies attached to the carboxyl groups of polystyrene NPs are fully functional [73].

The use of “dysopsonic proteins” is another way to build PCS. In this respect, molecular simulations and in vitro assessments have revealed an inverse relationship between the number and the distribution of hydroxyl groups on the surface of NPs and interaction with HSA/IgE but not for ApoE. Acting as dysopsonins, preadsorption of ApoE before injection enhances the blood circulation of NPs compared to their IgE-coated and pristine equivalent [74]. Trans blood–brain barrier (BBB) efflux of β-amyloid (Aβ) into the systemic blood stream is among the main ways for physiological clearance with ApoA1, ApoJ, and ApoE as chaperones. Taking advantage of this, bionic liposomes (SP-sLip) coated with a short peptide derivative of Aβ1-42 were constructed to cross the BBB for glioma therapy. This formulation resulted in a 45-fold increase in the distribution of SP-sLip relative to sLip in glioma mouse models [75]. Predefined with a hard protein corona, spherical nucleic acids (SNAs) are able to form stable PCs and tune NP behavior. When coated with anti-Her2 they can target specific cell types, while HSA coating avoids macrophage clearance (HSA), and engaging IgG results in different uptake machinery for the NP [76].

Although protein preadsorption has yielded promising results, there is no exact pattern to define NP action yet. For example, deposition of natural immunoglobulin on a biomolecular corona determines C3 complement opsonization of superparamagnetic iron oxide (SPIO) nanoworms and several clinically used NPs and may determine individual complement responses to nanomedicines [77]. However, complement activation in C3-deficient animals cannot explain differences in NP clearance [78]. Equally, surface hydrophilicity has been shown to be linked with lower or non-protein affinity of core-crosslinked polymeric NPs. The protein affinity of polysarcosine (pSar), poly(N-2-hydroxypropylmethacrylamide) (pHPMA), and PEG with prolonged circulation times and different hydrophilic polymer materials shows enrichment of some proteins with very low concentrations, such as less than one protein per particle [27]. Nonetheless, hydrophilicity may mediate the early clearance of different sized particles. However, PEG densities above a critical value (20 PEG chains (MW 5 ​kDa) per 100 ​nm) result in prolonged circulation times, irrespective of NP size. Additionally, studies on knockout mice show that the deposition of apolipoproteins on NPs with low PEG density can extend circulation times. A dominant pathway for nanoparticle clearance is through the low-density-lipoprotein receptor, regardless of PEG thickness [78]. These discrepancies can be rooted in several factors, such as NP design; however, the most important factor is tumor heterogeneity. That is, for each type of tumor (cell), the pattern of protein corona formation is completely different [28].

#### 2.1.4 Macrophage depletion

The removal of liver macrophages, the cells that take up the largest amount of circulating NPs, would lead to a significant increase in the NP systemic circulation. This strategy is attempted mostly by clodronate liposomes, which resulted in an improved NP delivery efficacy of only 2%. Intravenous injection of liposome-encapsulated clodronate can deplete peripheral macrophages, with an efficacy of reducing spleen and liver macrophages by >95% and blood monocytes by 55% [29]. Injection of clodronate liposomes 48 ​h prior to administration of 100 ​nm AuNPs can deplete splenic and liver macrophages, resulting in a prolonged half-life by 13 times, from 0.64 to 8.00 ​h, and increased AuNPs tumor delivery from 0.03 to 0.5% ID [79]. Injecting ‘decoy’ particles that eliminate plasma opsonins is another commonly used way of blocking RES uptake. As similar plasma opsonins can be absorbed to iron oxide and Ni^2+^, chelated Ni^2+^ was coated on the surface of liposomes to act as a decoy. Ni-liposomes resulted in a 5-fold increase in the half-life of intravenously injected SPIO and CREKA-SPIO NPs [80]. As an alternative to clodronate-encapsulated liposomes, biocompatible and safe RBC-derived nanovesicles (RDNVs) were adopted for clodronate delivery and specific macrophage depletion. Clodronate-loaded CD47 KO mouse-derived RBCs were more toxic against macrophages than CD47^+^ RDNVs (WT-RDNV/CLD), as they lacked the CD47 camouflage ‘don't eat me’ signal and thus were rapidly internalized by macrophages. Meanwhile, WT-RDNV/CLD showed prolonged tissue accumulation in the liver and lung, as well as macrophage depletion [81].

### 2.2 Engineering of the nanoparticles localization

The second important barrier is the vascular wall, which allows NP extravasation through blood vessels to exit systemic circulation and enter the tumor interstitium. This step determines NP localization and retention in the tumor region. NPs are believed to mostly possess the EPR effect of solid tumor vasculature. Thus, any strategy to enhance tumor permeability should be promising. However, given that only a mean of 0.7% of the injected dose (%ID) of NPs is delivered to cancer cells, the major role of EPR in NP uptake is questionable. A study on a subset of gold nanoparticles using computational modeling and simulation of physiologically based pharmacokinetics indicated that the low delivery efficiency largely stems from the low distribution and permeability coefficients at the tumor site [5].

#### 2.2.1 Vasodilation

Increased permeability to the tumor tissue through the vasodilation of vessels is an approach to treat cancer by facilitating oxygen transfer and suppression of hypoxia in the tumor. This can be attained by drugs or using physical methods such as ultrasound (US), radiotherapy (RT), thermal therapy, mild photodynamic therapy (PDT), or a cocktail of these physical methods combined with other interventions. For example, US-activated microbubbles can locally alter the TME through enhanced permeability, altered perfusion, reduced IFP, and reduced hypoxia [30,31]. Pulsed high-intensity focused ultrasound (pHIFU), as a US treatment approach, is particularly capable of improving tissue penetration and blood flow within the vasculature of the target organ, ultimately enhancing extravasation. In this line, pHIFU applied at 0, 10, 20, and 50 ​W can enhance the tissue penetration of fluorescent dye-labeled glycol chitosan NPs in the femoral tissue of mice by 0-, 5.7-, 8- and 9.3-fold, respectively [32]. It also enhances NP penetration, especially into the tumor core, by reducing IFP, expanding extracellular spaces by reducing collagen fibril content, increasing hydraulic conductivity, and decreasing tissue stiffness [82].

**Fig. 3:**
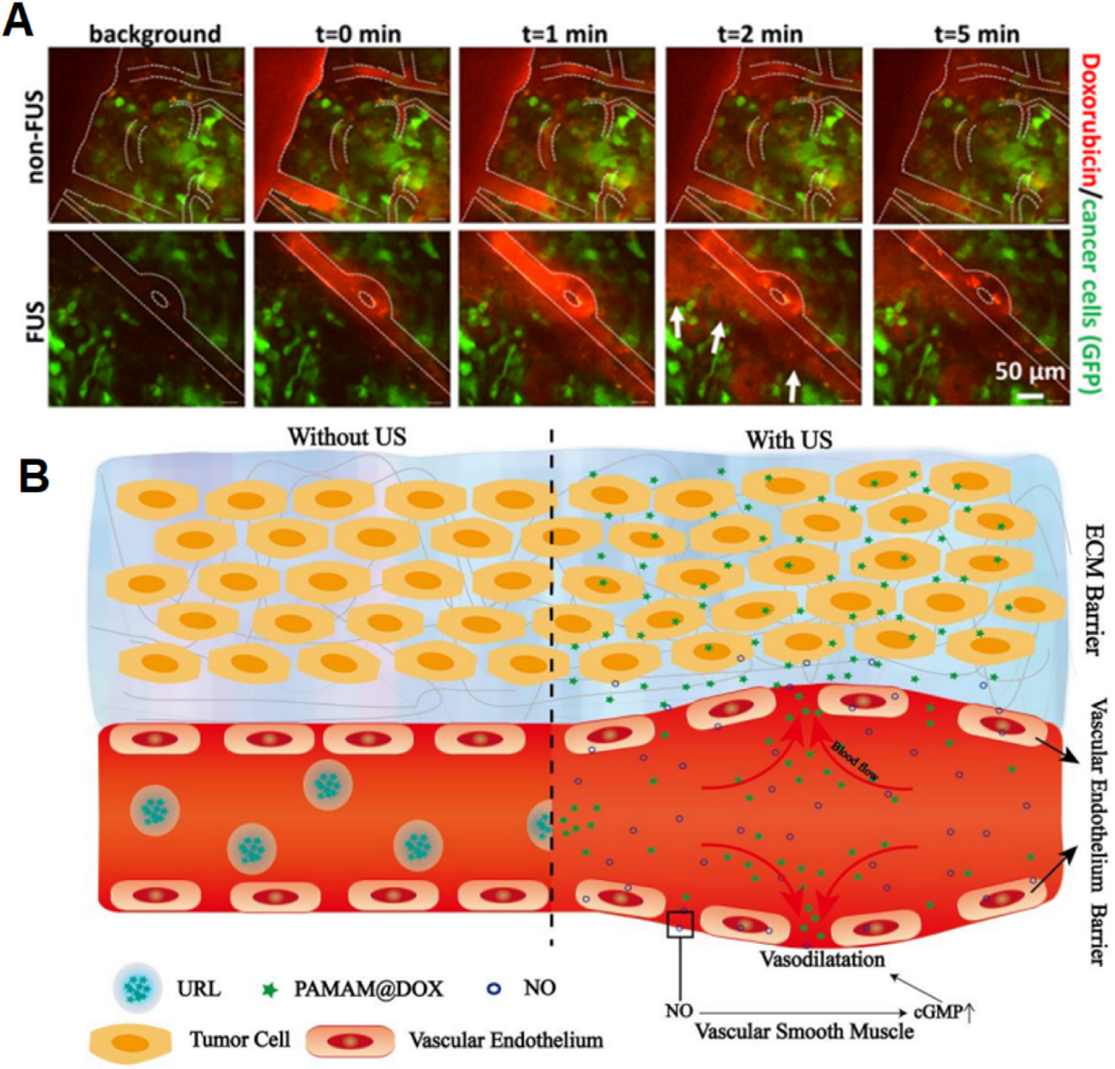
**FUS mechanism of action for improving EPR in solid tumors.** (A) Real macroscopic images of glioma tumors showing FUS-mediated disruption of the BBB, which enhances drug extravasation in BT474-Gluc metastatic brain tumors. Reprinted with permission [83]. Copyright 2018, National Acad Sciences (B) Cartoon animation of the combined work of US and NO therapy results in effective vascular barrier disruption for NP delivery. Reprinted with permission [84]. Copyright 2017, Elsevier.

Focused ultrasound (FUS) in combination with microbubbles is used for nontargeted delivery of DOX as well as targeted antibody–drug conjugates (for example, ado-trastuzumab emtansine (T-DM1)) to disrupt blood–brain/blood–tumor barriers (BBB and BTB) and enable interstitial transport in an orthotopic xenograft model of HER2-positive breast cancer brain metastasis. Extravasation showed a significant increase in drug penetration of FUS compared with non-FUS. Physiologically based pharmacokinetic (PBPK) modeling integrated with experimental data revealed that mechanistically, FUS combined with microbubbles increases hydraulic conductivity, which lessens vascular barriers and enhances interstitial convective transport. Additionally, a more than 2-fold increase in DOX transvascular transport through small vascular pores with diameters of 10–50 ​nm was achieved, as predicted by PBPK (Fig. 3A) [83].

Selective dilation of tumor blood vessels with nitric oxide (NO), which works on endothelial smooth muscle, can be used for promoting vascular permeability and enhancing NP accumulation at the same time. Hybrid polymeric micelles for targeting tumor vasculature are prepared that are coated with carboxylate PEG to afford micelle stability and a prolonged blood circulation half-life under neutral conditions. In mildly acidic tumor tissues, degradation of the PEG layer exposes cyclic Arg-Gly-Asp (cRGD) peptides, which bind to integrin receptors overexpressed on the tumor endothelium. Simultaneously, local NO generation can occur as exposed copper ions catalyze the decomposition of endogenous NO donors. The resultant vasodilation and increased EPR promoted DOX accumulation at 24 ​h after injection by 3.1-fold (15.1% ID/g) compared with 4.9% ID/g for Dox-loaded hybrid micelles without copper ions and 4.8% ID/g for micelles without cRGD [85]. Another strategy combines the reversible vasodilatory effect of NO with size-changeable ultrasound-responsive liposomes (URLs) capable of size switching to simultaneously extravasate the endothelial gap and ECM. Under US stimulation, fast NO generation occurs from donor S-nitrosoglutathione, which acts on the tumor's small muscle to promote tumor vascular dilation. At the same time, NO generation ruptures the URL lipid bilayer, releasing PAMAM-DOX NPs with a small size of ∼10 ​nm to deeply penetrate the tumor. The combination vasodilatory effect of NO and the size-controlled characteristic of URL allows a variety of drugs to extravasate through the endothelial gap (Fig. 3B) [84]. Another agent, sildenafil, a vasodilator ampholyte, is also released under tumor acidity and selectively promotes the intratumoral accumulation of nanomedicines by increasing the EPR effect [86]. Similarly, hydralazine (HDZ), an antihypertension vasodilator, has been shown to elicit dramatic effects on NP penetration by reducing the tumor stroma and as an adjuvant with particular application in the treatment of advanced desmoplastic tumors [87].

#### 2.2.2 Vascular normalization

**Fig. 4:**
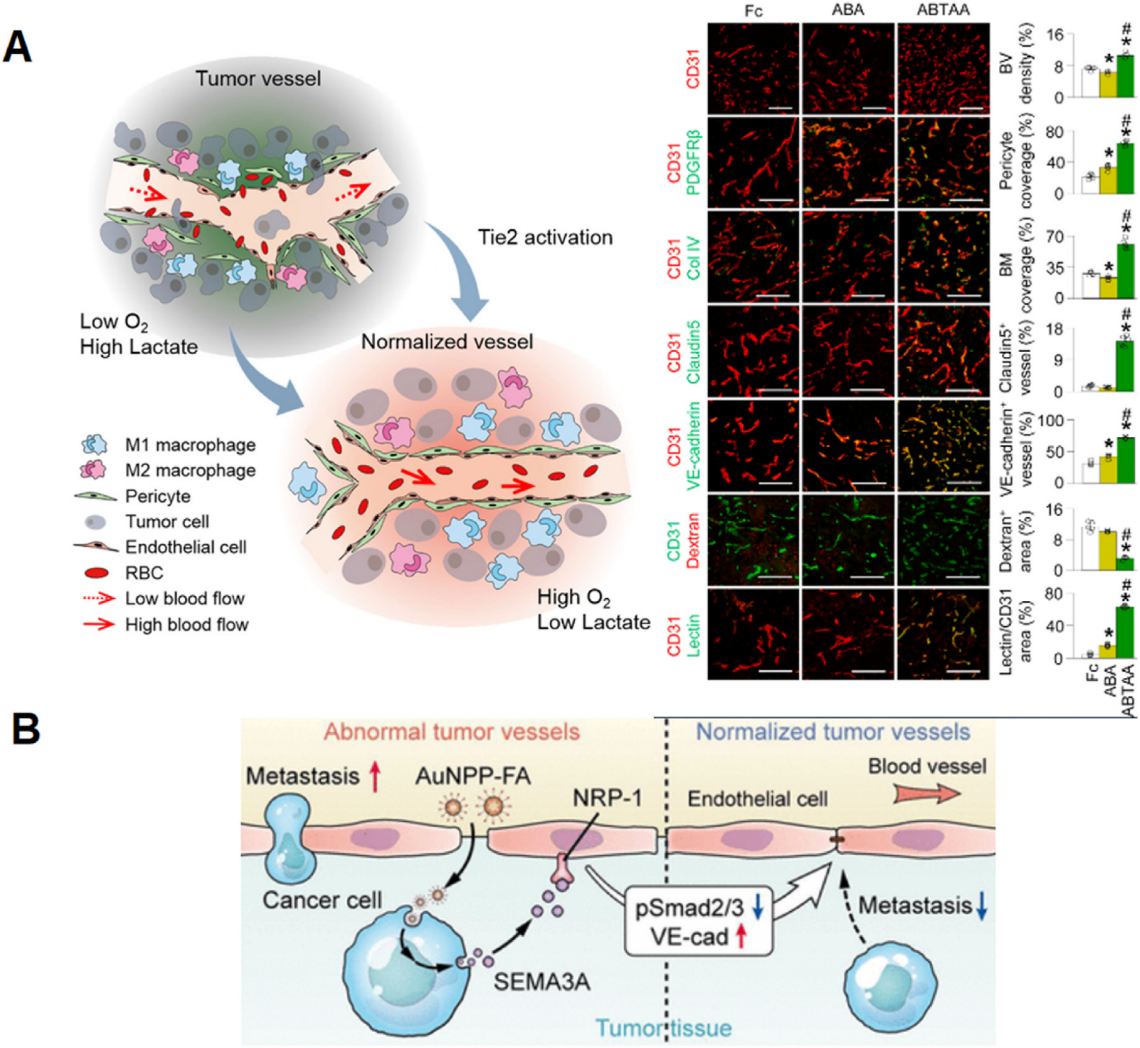
**Tumor vasculature normalization to cross the vascular barrier.** (A) Functional and structural changes in TEC markers occur during vascular normalization to promote enhanced blood perfusion in tumors for drug delivery. Reprinted with permission [92]. Copyright 2016, Elsevier. (B) Vascular normalization properties of Au NPs can inhibit tumor metastasis by closing vascular gaps. Reprinted with permission [93]. Copyright 2020, American Chemical Society.

Blocking the formation of new blood vessels, or anti-angiogenesis (AA), is a well-known tactic for vascular normalization and cancer therapy. Tumor ‘vascular normalization’ using antiangiogenic therapy can improve the delivery and effectiveness of nanomedicines [88]. One reason for this effect comes from a study in which suppressing the activity of TECs with hyper-glycolytic metabolism through blockade of the glycolytic activator PFKFB3 elicited anti-metastatic potential with improved vessel maturation and perfusion by ‘tumor vessel normalization’. Anti-PFKFB3-mediated glycolysis reduction resulted in a stiffened vascular fence by decreasing vascular endothelial cadherin (VE-cadherin) endocytosis in TECs, rendering pericytes more dormant and adhesive (highly positive for N-cadherin expression) and downregulating NF-κB signaling, which reduced cancer cell adhesion molecules in TECs [89]. It is worth noting that the aforementioned principle is a working mechanism for the toxicity of some small NPs, where small NPs can rapidly accumulate in and interact with biological tissues. In fact, this interaction in the case of small titanium oxide (TiO_2_) NPs was shown to cause enhanced tumor leakiness by disrupting the homophilic interaction of VE-cadherin, increasing the number of pulmonary metastases in a melanoma-lung metastasis mouse model. More precisely, TiO_2_ results in the phosphorylation of VE-cadherin at intracellular residues and the loss of its interaction with p120 and β-catenin. This promotes actin remodeling followed by VE-cadherin internalization and degradation [90]. These effects can be counteracted using the anti-permeability growth factor angiopoietin-1, which accelerates the restoration of endothelial cell barrier integrity from NP-induced leakiness by forming complexes with its receptor Tie2 at cell-to-cell junctions [91]. A strategy involving Tie2 activation and Ang2 inhibition promotes a favorable TME for enhanced chemotherapeutic (e.g. temozolomide) delivery through enhanced blood perfusion in mice with spontaneous mammary cancer, subcutaneously implanted Lewis lung carcinoma and orthotopically implanted glioma. ABTA (Ang2 binding and Tie2 activating antibody) treatment resulted in enhanced blood perfusion, lessened hypoxia, lactate acidosis, brain edema, and enhanced chemotherapeutic drug delivery. ABTAA reduced dextran leakage by 80%, while it markedly promoted vascular perfusion (Fig. 4A) [92]. Thus, vascular normalization strategies that either block the metabolism of TECs or ABTA can enhance NP drug delivery by crossing the transvascular barrier. Of note, the same effects have also been attributed to Au NPs. Recent data show that Au NPs can elicit tumor vessel normalization, as evidenced by increased pericyte coverage and increased VE-cadherin tight junctions. In this way, folic acid-functionalized Au NPs normalize vessels and inhibit metastasis by inducing the secretion of semaphorin 3A to block endothelial Smad2/3 signaling in vitro (Fig. 4B) [93].

With respect to the effect of vascular repair following anti-vascular endothelial growth factor receptor-2 (VEGFR2), studies indicate an effect of NP size on the efficacy of nanodrug delivery, with positive effects on the delivery of small NPs (12 ​nm) and negative impacts on larger 125 ​nm NPs. Mathematical modeling has shown that reducing tumor leakiness and IFP is achievable by lowering the vascular wall pore sizes, favoring the rapid penetration of small NPs. In addition, penetration of larger NPs is retarded through smaller pores due to increased steric and hydrodynamic hindrances [33]. Similarly, to normalize the ECM and the tumor vasculature, anti-transforming growth factor β1 (TGF-β1) antibody and DC101, an anti-vascular endothelial growth factor receptor antibody, have been used in a syngeneic murine model of glioma. This improved the structured vascular network, enhanced tissue perfusion, and decreased collagen density, together improving the delivery and distribution of the nanoparticle within the tumor [94]. Wei et al. developed a novel strategy combining cediranib, a tumor vessel normalization agent, with an enzyme-responsive size-changeable gold nanoparticle to treat cancer. Results showed the co-administration of cediranib and AuNPs-A&C achieved prevailing tumor targeting and antitumor efficacy in the 4T1 tumor-bearing mouse model [95].

#### 2.2.3 Vascular disruption

Vascular disruption induced by vascular disrupting agents (VDAs) is another way to achieve vascular normalization capabilities. Unlike anti-angiogenesis therapy, which aims for neovascularization and the formation of new blood vessels, vascular disruption targets pre-established tumor blood vessels. VDAs can produce more than 90% central necrosis in bulky tumors but no complete tumor remission, as they mostly leave a thin layer of residual cells (rim cells) [34]. To produce long-lasting effects, VDAs are preferably coupled with other anticancer modalities or encapsulated in nanoformulations to improve their bioavailability and half-life. As a leading VDA agent, combretastatin A4 (CA4) works by reversible tubulin disruption (microtubule inhibitor). Unlike actin, which is dominant in the cytoskeleton of healthy, mature vasculature, microtubules form the cytoskeleton of new blood vessels and CA4P cytotoxicity is therefore restricted to tumors with immature vessels. Moreover, normal vessels should have a flat structure to keep the lumen open for blood circulation. Microtubule inhibition makes tumor endothelial cells (TECs) rounder, occluding the blood lumen and reducing blood flow, followed by hemorrhage, hypoxia, and finally tumor cell necrosis. Additionally, CA4P can induce apoptosis by interrupting microtubule polymerization and mitosis [35].

Although high tissue penetration in solid tumors is prescribed for the effectiveness of most nanomedicines, for CA4P to elicit long-lasting vascular disrupting effects, a nanoformulation designed to encourage a higher vascular rather than tissue accumulation of the drug is necessary. Along this line, polymeric NP composed of poly (l-glutamic acid)-CA4 conjugate (PLG-CA4) has been used to enhance CA4 accumulation and retention in tumor tissue. The results demonstrated the advantages of the CA4 nanodrug conjugate, as it allowed high dissemination and slow release of CA4 focused on tumor blood vessels. This promoted sustained vascular disruption and markedly improved the therapeutic index by 74% for PLG-CA4 versus 24% for CA4P in vivo [96]. Furthermore, CA4P administration after NP therapy can promote enhanced EPR and increase NP accumulation and retention. For example, CA4P injection 3 ​h after NP therapy was shown to significantly slow the growth of aggressive large desmoplastic tumors by causing massive central apoptosis and simultaneously increasing radiation-containing NP uptake in the tumor that lasted over several days. It was surmised that the heightened NP accumulation was affected by CA4P action to induce hemorrhage; as wide distribution of RBCs in the tumor interstitium was evident, NPs with smaller sizes should have the same opportunity to flow through broken vasculature deep into the tumor region. In addition, at earlier stages, CA4P action promotes NP retention and localization by inducing vascular blockade and congestion [36]. Moreover, selective aggravation of hypoxia related to CA4P therapy is used for enhanced activity of hypoxia-activated prodrugs (HAPs) like Tirapazamine for the treatment of metastatic tumors [37].

**Fig. 5:**
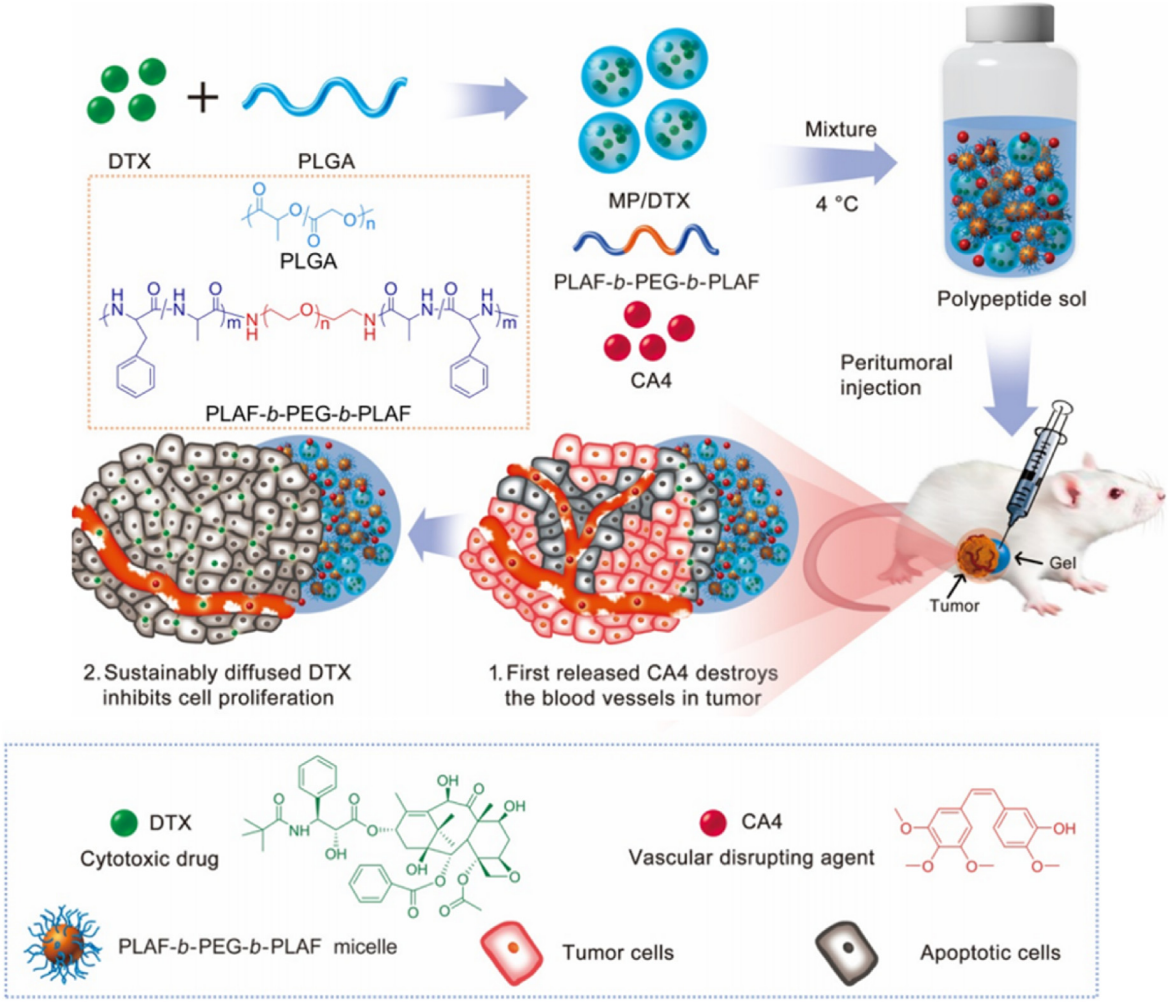
**Tumor vascular disruption for enhanced NP delivery to tumor cells.** The preparation and mechanism of action of Gel/CA4−MP/DTX for sequential drug delivery and locally synergistic chemotherapy of osteosarcoma are depicted. Reprinted with permission [38]. Copyright 2018, Elsevier.

Other formulations based on ‘multistage systems’, which allow for the sequential release of different drugs from NC called the ‘one-tablet formulation’, are also in development. PVP/PLGA and PCL/PLGA NPs with a distinct core–shell structure are used for loading the hydrophobic CA4 and the hydrophilic drug (DOX) separately in the inner and outer compartments to realize ‘sequential drug release’ profiles in vitro [97]. To further translate this feature into an in vivo system, CA4P and docetaxel (DTX)-loaded microspheres embedded in an injectable thermosensitive polypeptide hydrogel (Gel–MP construct) have been employed for the synergistic treatment of osteosarcoma. CA4 release occurs faster to induce vascular disruption and cut off the tumor nutrient pathway connected to other tissues. Thus, it creates an interstitial space in the tissue for DTX penetration to efficiently suppress mouse K7 osteosarcoma growth in vivo (Fig. 5) [38].

Radio-sensitizing Au NPs acting as VDAs are used to specifically amplify damage in the tumor neoendothelium when applied in the treatment of intransigent or nonresectable tumors that respond minimally to standard therapies [39]. To examine the photothermally-enhanced vascular disrupting effects of gold NPs, DMXAA has been used as another potential standard VDA for fibrinogen aggregation. In this study, induction of thrombosis by later DMXAA injection was used to assemble fibrinogen-conjugated AuNPs (fAuNPs) into insoluble clots in tumor vessels, which was followed by NIR irradiation to promote photothermal ablation of tumor vessels. DMXAA injection resulted in rapid NP accumulation, resulting in 2.6- and 2.1-fold higher intratumoral fAuNP contents at 12 ​h and 24 ​h, respectively, than that of control groups without DMXAA treatment [98].

#### 2.2.4 Vascular infarction

Distinct from vascular disruption, vascular infarction involves selective induction of vascular thrombosis in the tumor-feeding endothelium and thus fibrin clot formation. Ideally, the induction of thrombosis should be restricted to the tumor. This can be achieved by decorating the NPs with TEC-specific markers. Rather than just pursuing targeted delivery of NPs to induce local thrombosis, coagulation-based self-amplifying systems inspired by platelet propagation employ biomimetic particles that not only home to tumors but can amplify their own localization. The system is based on a CREKA peptide that binds coagulated plasma proteins, particularly fibrin clots on vessel walls and tumor stroma. CREKA-coated iron oxide NPs and liposomes home to tumor vessels, where they locally activate coagulation, thus producing additional sites for binding of more NPs. Coagulation contributed to an approximately 6-fold greater accumulation of CREKA-SPIO in the group pretreated with decoy NPs than in the PBS-pretreated mice. This pretreatment enhanced NP accumulation, as evidenced by a more than 50% drop in CREKA-SPIO tumor accumulation in the presence of heparin, a strong inhibitor of clotting [80].

Another coagulation-based self-amplification strategy is called the ‘two-component system’, which is composed of ‘signaling’ and ‘receiving’ modules and is based on communication with different NPs. The signaling component includes heatable gold nanorods or recombinant tissue factor (tTF-RGD), which, upon homing to tumors, induce in situ clot formation. This broadcasts a coagulation cascade, and more precisely the tumor site, which is characterized by accumulation of clotting factors including transglutaminase factor XII and fibrin, to CREKA-targeted ‘receiving’ nanoparticles (magneto-fluorescent iron oxide nanoworms and DOX-loaded liposomes) in circulation. This autonomous self-amplification biomimetic platform resulted in the localization of 40-fold higher doses of chemotherapeutics to tumors than non-communicating controls [99]. Furthermore, a tubular DNA nanorobot has been fabricated that bears a nucleolin-binding DNA aptamer on the surface and a thrombin protein inside the cavity. Nanorobots can selectively bind to TECs through the recognition of nucleolin. This results in the mechanical opening of the DNA nanorobot to expose thrombin and activate tumor-site-specific coagulation in mice and Bama miniature pigs. Targeted therapy resulted in a 7-fold increase in nanotube accumulation compared with the nontargeted nanorobot 8 ​h after injection [100].

**Fig. 6:**
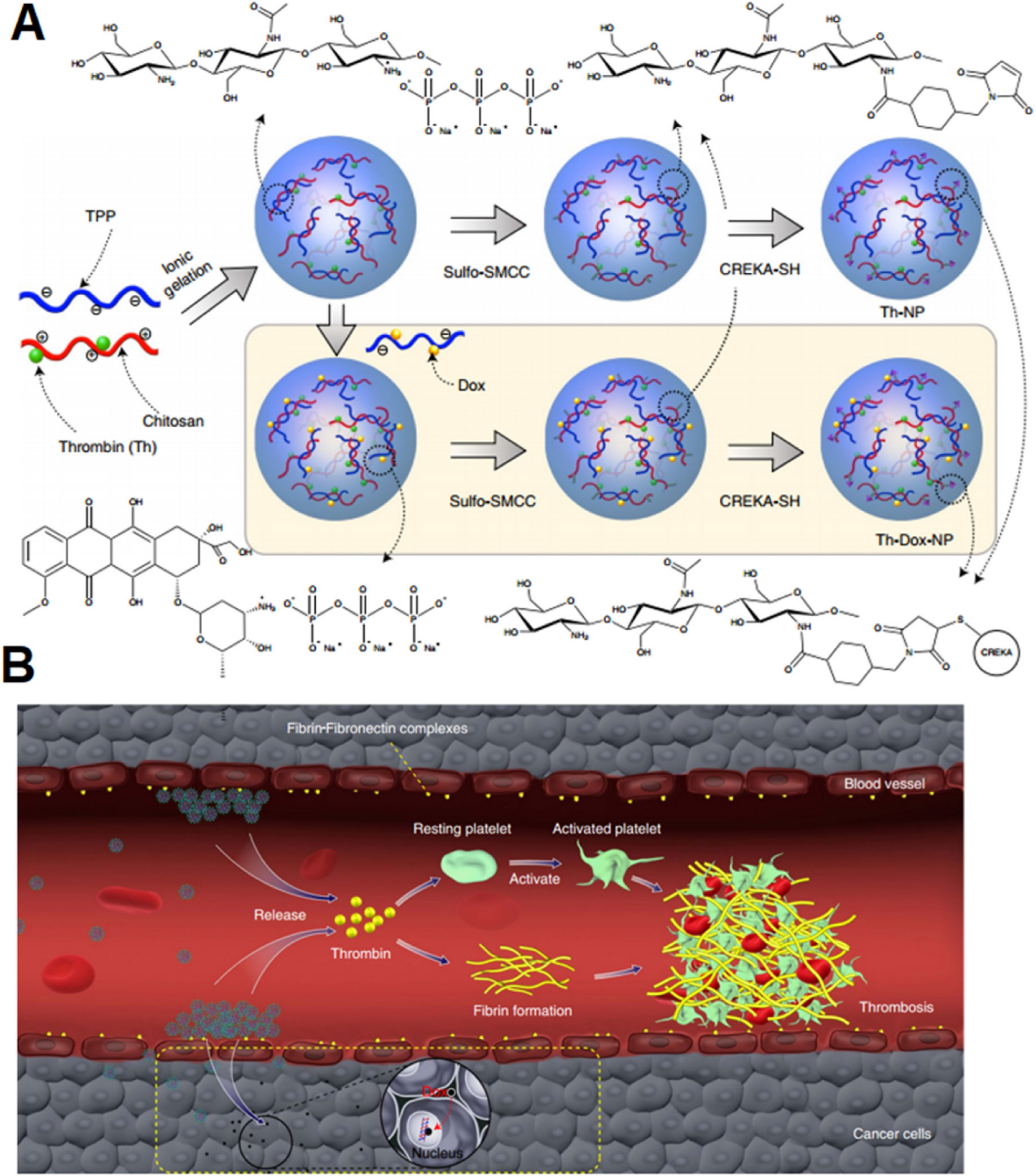
**Tumor vascular infarction.** (A) Schematic of encapsulating thrombin and Dox and making chitosan-based polymer NPs. After ionic gelation, NPs were surface functionalized with sulfo-SMCC to supply the maleimide group for the Michael addition process with the CREKA peptide. (B)NP activity in the tumor. When CREKA contacts fibrin-fibronectin complexes inside tumor arteries, the NPs release thrombin to create a thrombus, while Dox (black dots) kills tumor cells. Reprinted with permission [101]. Copyright 2020, Nature Publication Groups.

Furthermore, the same group combined the concept of tumor infarction therapy with chemotherapy to achieve a prolonged anticancer therapeutic index. In the new study, CREKA that was decorated on the surface of DOX and thrombin-loaded chitosan nanoparticles (Th-Dox-NPs) was used to home NPs to fibrin–fibronectin complexes found abundantly in the tumor stroma and tumor vessel walls. Systemic injection of NPs into mice and rabbits bearing subcutaneous or orthotopic tumors resulted in significant tumor growth inhibition, with 80% complete tumor ablation in mice lasting for >45.0 days after treatment cessation. CREKA-targeted NPs showed 28-fold more accumulation in the tumor than nontargeted particles 8 ​h after administration. The in vivo half-lives of Dox for targeted and nontargeted NPs were 4.1 ​h and 1.6 ​h, respectively. There was a 2.8-fold drop in the nonspecific accumulation of Th-Dox-NPs in the heart compared with free-drug administration at the equivalent dose (Fig. 6) [101].

**Fig. 7:**
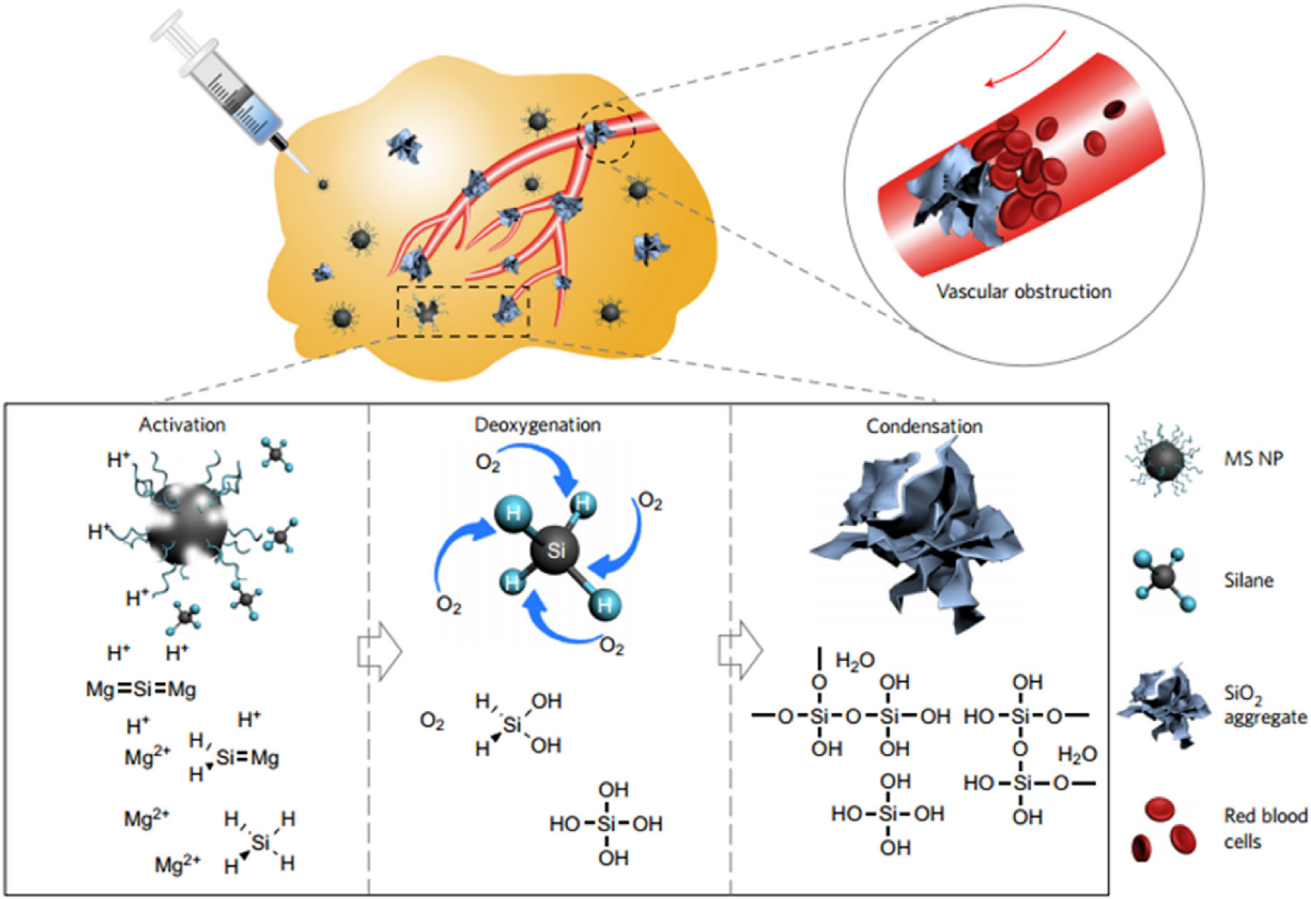
**Tumor deoxygenation for starving tumor cells.** MS NPs are intratumorally injected for cancer-starving therapy. The tumor blood capillaries produce in situ SiO_2_ blockers to deoxygenate tissue and prevent reoxygenation after the acidic tumor microenvironment activates NPs. Deoxygenated tumors suffocate without energy metabolism. (MS NPs are PVP-modified). Reprinted with permission [40]. Copyright 2017, Nature Publication Groups.

Rather than using biological coagulation factors, vascular infarction can also be chemically induced. Polymer-modified magnesium silicide (Mg_2_Si) nanoparticles have been used as deoxygenation agents and as materials that form anti-capillary agents through their byproducts, additionally hampering the tumor from being reoxygenated. Silane is released from Mg_2_Si under the acidic microenvironment of the tumor and reacts with oxygen, be it tissue-dissolved or bound to hemoglobin, forming silicon oxide (SiO_2_) aggregates. Ultimately, SiO_2_ is capable of blocking blood capillaries in the TME, thus preventing oxygen and nutrient supply from reaching the tumor. The SiO_2_ network can work as a signaling module that locally induces coagulation without using peptide-homing moieties (Fig. 7) [40].

#### 2.2.5 Active vascular targeting

Following analysis of different types of human and mouse tumors plus mathematical modeling, simulation, and imaging techniques, a study has criticized the 30-year concept of EPR, which is regarded as the most important pathway for NP delivery to solid tumors. The findings of this study suggest that NP transport into solid tumors is not mediated by inter-endothelial gaps (EPR). Instead, endothelial cells are used actively by up to 97% of NPs to enter tumors. The primary method of NP extravasation into tumors is through *trans*-endothelial pathways [102]. Thus, EPR should be attempted along with active targeting strategies, mostly achieved by the functionalization of tumor-specific targeting moieties.

**Fig. 8:**
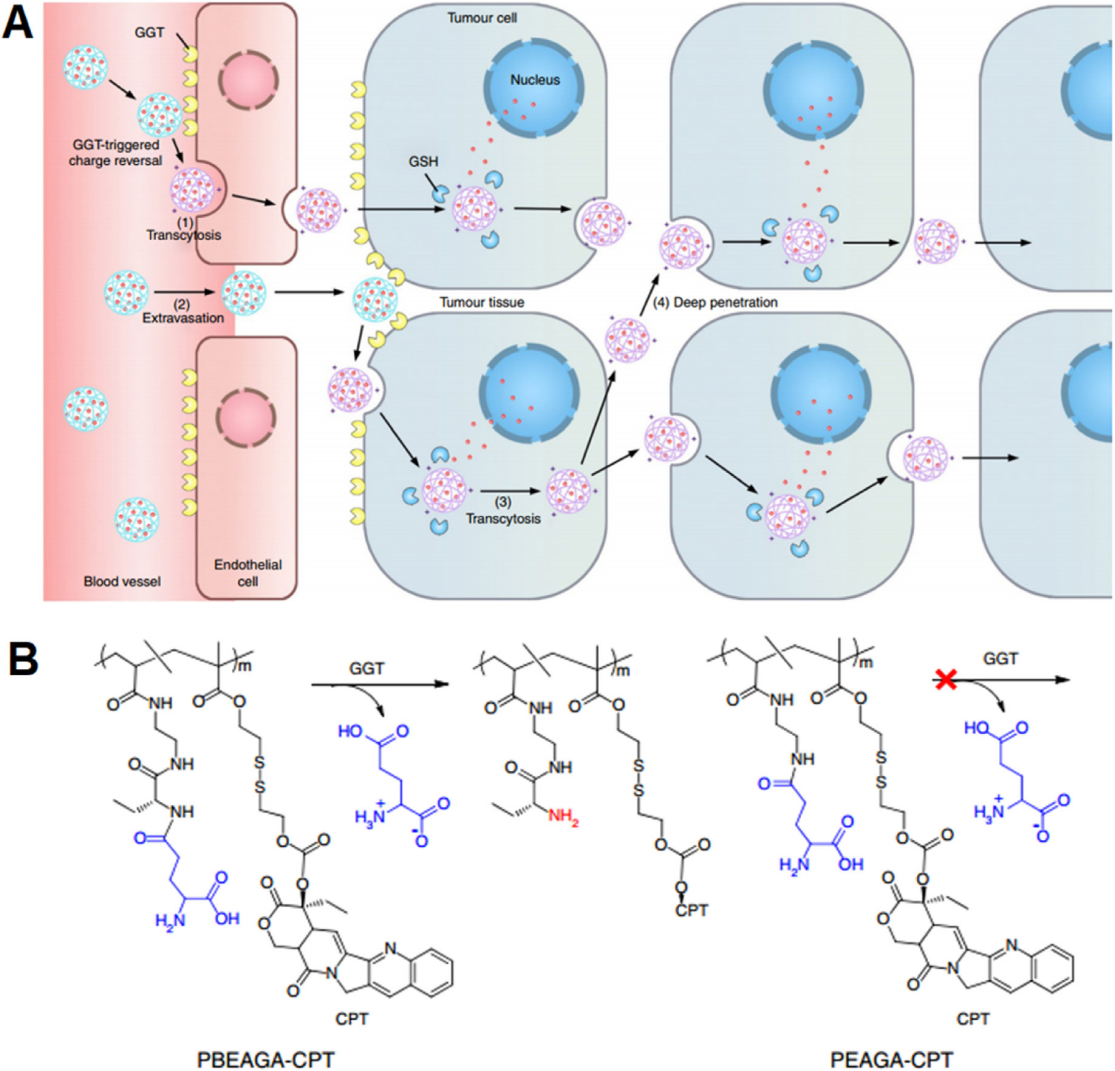
**Enzyme-based controlled charge-changing principle for prompting active transport of nanomedicines through trans-endothelial and transcellular barriers.** (A) Schematics of working principle. (B) Cationization reaction of enzyme-activatable polymer–drug. Reprinted with permission [103]. Copyright 2019, Nature Publication Groups.

For example, a positively charged NP, although reducing its blood circulation, is desirable for crossing the transluminal endothelium/intratumoral lining and can favor ATP-mediated active caveolae-mediated transcytosis. To achieve both features at once, an enzyme-responsive camptothecin–polymer conjugate is harnessed to afford a charge-changing feature for NP that is programmed to occur specifically at the interface of the endothelial cell membrane, which expresses high amounts of a γ-glutamyl-trans-peptidase. This enzyme digests the γ-glutamyl moieties of the conjugate, producing a cationic NP with primary amines to enable passage and penetration of the resultant positively charged conjugate through active as well as passive *trans*-endothelial and *trans*-cellular transport. Achieving homogenous diffusion across the tumor region, this formulation has been 100% effective in ablating small tumors and effective in inhibiting the growth of large orthotopic pancreatic solid tumors in mice (Fig. 8) [103].

Another strategy to breach the vascular barrier is through tumor-penetrating peptides (TPPs). For one, iRGD administration is used to enhance transcytosis of silicasome-loaded irinotecan through the neuropilin-1 (NRP-1) pathway for the treatment of pancreatic ductal adenocarcinoma (PDAC). Ultrastructural imaging studies using Au-labeled silicasomes (lipid bilayer–coated MS NPs) revealed that free iRGD co-administration induced a vesicular transport pathway, transporting NPs from the blood vessel lumen to a perinuclear site within cancer cells, resulting in 3-4-fold uptake of NPs in a KPC-derived orthotopic PDAC model and noticeable suppression of tumor growth and metastasis [104].

#### 2.2.6 Vascular component depletion

Rather than activating platelets, as described for ‘self-amplifying systems’, the opposite strategy works by depletion of serum/stroma cells. For example, protease matrix metalloprotease 2 (MMP2)-responsive polymer–lipid–peptide NPs encapsulating DOX and antiplatelet antibody R300 (PLP-D-R) have been fabricated, wherein R300 release can be triggered by the presence of MMP2, which is abundantly expressed in tumor vascular endothelia and stroma. Further antiplatelet activity locally depletes tumor-associated platelets, thereby augmenting EPR and enhancing NP accumulation in tumors with substantial metastasis inhibition and tumor regression in mice. Using PLP–D–R, the elimination half-life and distribution half-life of Dox were calculated to be 58.36 ​h and 2.05 ​h, respectively, while an equivalent dose of free DOX resulted in only 19.40 ​h and 0.89 ​h, indicating the prolonged in vivo retention of drug cargoes by their formulated NPs [105].

#### 2.2.7 Trojan system

Inspired by the ancient ‘Trojan horse’, cellular, acellular, or chemical carriers are constructed that are harmless in appearance, but contain toxic cargoes to be deployed specifically upon entrance into their designated hosts (tumor cells). Living cells such as MSCs, monocytes, macrophages, and bio-organisms such as bacteria could be employed as natural carriers and Trojan horses for specific cargo delivery to tumor regions. Possessing an intrinsic homing property, they have the ability to cross physiological barriers and reach disease sites. MSCs possess an inherent tropism to malignant cells as well as homing to disseminated tumor foci; thus, they have been proposed as ideal targeting vehicles for various purposes in drug delivery [41]. Likewise, similar intrinsic properties have been demonstrated to exist in immune cells, making them promising candidates for tumor-targeted therapy [106].

In the case of cancer immunotherapy, the significance of immune cells as Trojan horses and for NP efficacy is apparent. Accordingly, recent data have shown that the retention of antibody-labeled 100 ​nm hydroxyethyl starch–coated iron oxide nanoparticles in tumors is not determined by antigen-antibody interactions; rather, it relies on NP interactions with tumor-associated monocytes, macrophages, neutrophils, and dendritic cells. Systemic exposure to either nanoparticle type can induce T-cell–mediated tumor suppression even in the absence of target antigen in breast cancer models in mice, suggesting that NP interaction with immune cells dominates tumor retention [107]. Another study showed the importance of the neutrophil-mediated route for the delivery of rapidly cleared NPs. The results indicated that the efficiency of Trojan horse-mediated NP accumulation varied in different tumors, with more efficacy in neutrophil-rich tumors [24].

As the first example of Trojan horses, MSCs are used for selective photodynamic therapy (PDT) of cancer. To this end, drug-loaded hollow silica NPs are internalized into MSCs to carry and deliver a photosensitizer drug to breast tumors. The MSCs with high tumor affinity were used to overcome the challenges associated with the specific accumulation of photosensitizer drugs in tumors for effective PDT [108]. With the aim of developing a systemically acting Trojan horse platform for targeting metastatic prostate cancer, poly (lactic-*co*-glycolic acid) (PLGA) microparticles (MPs) containing the G114 prodrug were loaded into human mesenchymal stem cells (hMSCs). G114-particles (∼950 ​nm in size) loaded in MSCs were capable of sustained release of G114 as an intact prodrug for 7 days and selective activation of cell death in the prostate specific antigen (PSA)-secreting prostate cancer cell line LNCaP in vitro and tumor growth inhibition in vivo [109]. MSCs loaded with chlorin e6-conjugated polydopamine NPs (MSC-PDA-Ce6) were able to accumulate and deeply penetrate the lung tumors of mice with lung melanoma metastasis. The Trojan system allows a cycling process of endocytosis–exocytosis–endocytosis among MSCs and cancer cells for enhanced dual PDT/PTT cancer therapy [41].

With the ability to home to sites of disease, many immune cells can eradicate abnormal cells and infections and can also be used for the delivery of therapeutics into tumors. For example, Ly6C^hi^ monocytes were loaded with paclitaxel (PTX)-encapsulating pH-sensitive micelles (PM) referred to as PM@MC. PM@MCs were capable of homing to primary tumors and the foci of lung metastasis in vivo, where they exerted greater PTX accumulation than commercial PTX injections and exhibited 96.8% and 99.2% efficacy for tumor inhibition and lung metastasis suppression, respectively [110].

Many nanotherapeutics have failed to target metastases due to the poor penetration nature of overt metastases and the absence of the EPR effect in micrometastases. One approach addresses this issue by exploiting inflammatory monocytes, which are shown to be preferentially recruited to the metastatic niche, where they turn into mature macrophages (tumor-associated macrophages (TAMs)). Protease (legumain)-responsive nanoparticles were loaded into inflammatory monocytes for active targeting of lung metastases and initiated metastasis-specific release of the drug (mertansine) upon their differentiation. Due to their natural metastasis-homing abilities, the bioinspired system afforded deep penetration and drug delivery with 77.8% lung metastasis inhibition of 4T1 breast cancer cells [111].

**Fig. 9:**
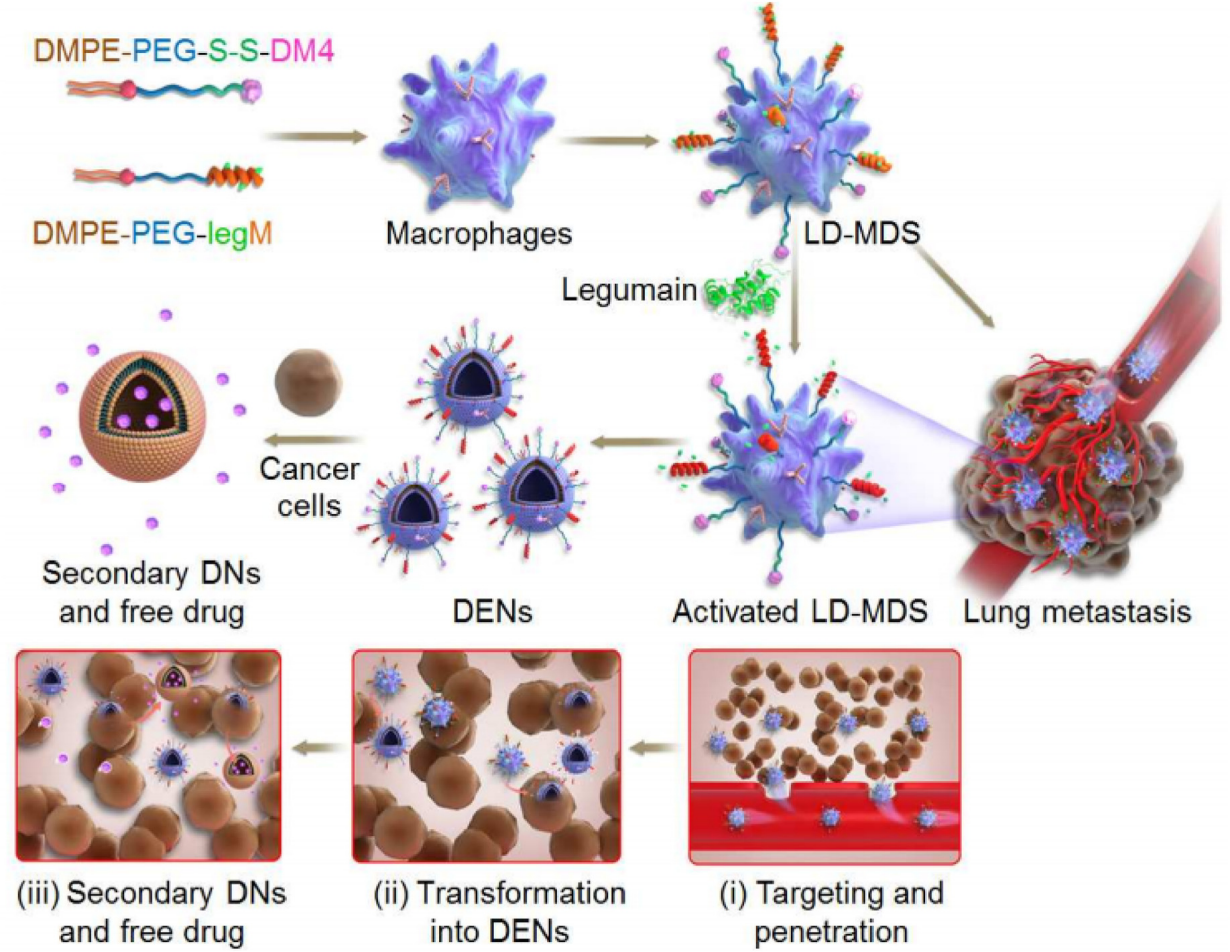
**Trojan system for crossing the vascular barrier and NP delivery.** Making LD-MDS, turning it into nanovesicles with legumain protease, and targeting it for lung metastases. LD-MDS preferentially are applied for lung metastases and converts into nanovesicles and secondary nanovesicles for successful antimetastasis therapy. Reprinted with permission [112]. Copyright 2018, American Chemical Society.

Another approach takes advantage of macrophages by developing a bioengineered macrophage-based delivery system (LD-MDS) capable of preferential drug delivery to lung metastases through transformation into nanovesicles and secondary nanovesicles. The membrane of living macrophages was altered by anchoring leumain-sensitive pro-peptide as well as the cytotoxic soravtansine (DM4) prodrug. When activated, the LD-MDS is transformed into exosome-like nanovesicles, which may facilitate efficient internalization by metastatic cancer cells. Subsequently, damaged cancer cells would release secondary nanovesicles, destroying adjacent cancer cells (Fig. 9) [112]. Furthermore, tumor-tropic monocytes/macrophages combined with remote-controlled FUS-triggered chemotherapy drug liberation have been reported for targeting therapeutics into hypoxic cells. While NPs are limited to a depth of 10–15 ​μm, hypoxia-tropic monocytes allow the delivery and accumulation of drugs to a depth beyond 150 ​μm from the adjacent blood vessels [113].

Additionally, engineered neutrophils responsive to inflammation have been used for live tracking of the internalization of DOX-loaded magnetic mesoporous silica nanoparticles (ND-MMSNs) by magnetic resonance (MR) imaging. Therefore, actively targeting inflamed brain tumors is feasible. Not only phagocytized D-MMSNs show high drug loading efficacy, but they also do not affect the viability of the host neutrophils, together increasing the intratumoral concentration of the drug and delaying relapse of surgically removed glioma [114]. Another recent work exploited neutrophils for the delivery of systemically delivered PTX-PLGA NPs, which promoted their in vivo self-armed assembly into circulating neutrophils, while CXCL1-laden hydrogels preimplanted in tumors recruited neutrophil-laden NPs to the tumor site through chemotactic effects [115].

In another preparation, chemotaxis-driven recruitment of neutrophils to inflamed tissues (cancer) was adopted for the effective crossing of various barriers, particularly the tumor vascular-interstitial barrier. This system employs bacteria-secreted outer membrane vesicles (OMVs) on PEG-b-PLGA micellar NPs. Then, pathogen-mimicking nanopathogenoids (NPNs) are used to hitchhike on circulating neutrophils. This formulation increased the tumor accumulation of NPNs by 2-fold compared with EPR-mediated NP delivery. Strikingly, PTT pretreatment of the tumors drastically augmented the tumor infiltration capability of neutrophils by ∼300–600%, even in the low-perfused blood vessels located in the core of solid tumors. Compared to monocytes and other types of cells, 83.4% of the total NPNs were internalized by neutrophils. A single PTT treatment plus NPNs@Pt resulted in 97% tumor growth inhibition and 60% complete tumor eradication in mice [116].

**Fig. 10:**
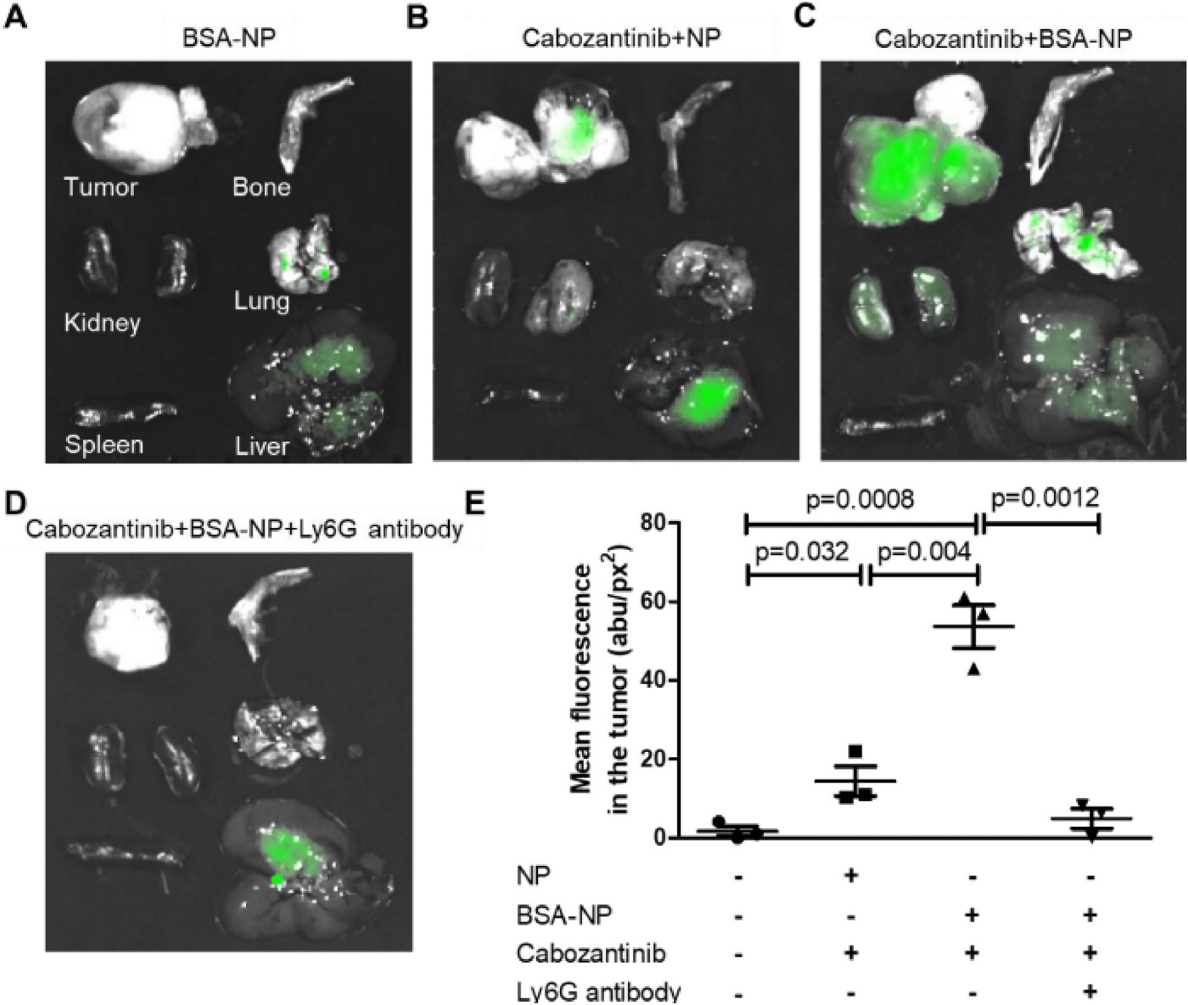
**Neutrophils are Trojan systems for the tumor-targeted delivery and localization of NPs**. In vivo, cabolantinib increases neutrophil-associated, dye-loaded BSA-NPs in prostate cancers. Fluorescent images were overlaid with bright field images used for treatment groups (A) BSA-NP, (B) cabozantinib ​+ ​uncoated NP, (C) BSA-NP, and (D) BSA-NP ​+ ​Ly6G antibody (neutrophil depletion). (E) Tumors were identified on superimposed fluorescence/bright field images using ImageJ, and the mean fluorescence was calculated to estimate NP uptake. Reprinted with permission [117]. Copyright 2021, American Association for Cancer Research.

Interestingly, pretreatment with some agents, such as cabozantinib, a tyrosine kinase inhibitor, can trigger neutrophil-mediated NP accumulation in tumors. Pretreatment with cabozantinib resulted in a 4-fold increase in neutrophil-associated BSA-coated, dye-loaded NPs that were systemically injected into prostate-specific PTEN/p53-deficient mice. By Day 3, 1% (pretreated with cabozantinib) and 0.11% (no treatment with cabozantinib) systemically injected dye-loaded NPs selectively accumulated in tumors. Strikingly, Ly6G (neutrophil-depleting antibody) completely abolished NP percolation in tumors, demonstrating the key role of neutrophils in targeted NP delivery to the prostate cancer stroma (Fig. 10) [117].

Tumor-tropic bacteria can also be used as cellular carriers for the specific accumulation of drugs. In line with this, one study reported two strategies for bacteria-mediated NP delivery to hypoxic tumors. The first approach (cargo-based) hitchhike NP on the surface of the vegetative *B. breve* while the second approach (antibody-based), requires injection of *C. difficile* spores first, then the antibacterial antibody-NP conjugates are administered to specifically target the germination of the *C. difficile* spores. Both approaches were designed to deliver upconversion nanorods for imaging and Au nanorods for NIR-induced photothermal ablation. The antibody-directed strategy showed the most intensive imaging signals, longer retention (∼6.6-fold), and the ability for complete tumor removal [118].

Apart from whole live cells, acellular components like cell-membrane vesicles (e.g., exosomes) and chemical components (NPs) can be exploited to act as Trojan systems. Exosomes prepared from different types of cells, mostly cancer cells, can direct drug payloads (e.g. Doxil [42], ZnO nanocrystal [119]) to their cancer cell of origin with high efficacy and precision. Additionally, supramolecular polylactide NPs acting as chemical Trojan systems can harbor DOX and release it controllably upon pH changes in the tumor milieu [43]. Unlike salt, which is non-toxic, an interesting study has shown the capability of NaCl NPs (SCNPs) as an effective Trojan system for ion delivery to cause osmolality perturbation to induce cancer cell death. This comes from the endocytosis-mediated transport of SCNPs, which redirects cell regulation of ion transport; thus, millions of Na^+^ and Cl^−^ ions are released inside cells upon the dissolvation of SCNPs. The opposing osmotic gradients across the plasma membrane trap these ions inside cells, leading to increased osmolality and rapid lysis of cells, while normal cells with relatively low sodium levels can resist these changes. Unlike conventional chemotherapeutics, SCNPs boost anticancer immunity in addition to toxicity to cancer cells in vivo [120].

### 2.3 Engineering of the nanoparticles penetration

Next to the vascular wall and the EPR effect is the ECM barrier, which determines NP localization and, in particular, penetration and distribution in tumor regions. Due to leaky vasculature, stiff ECM and lack of efficient lymphatic drainage, interstitial fluid pressure (IFP) in tumor tissue is significantly higher than that in normal tissue. This substantially diminishes NP retention, as it hampers NP diffusion in the tumor site. Thus, enhancing vascular permeability should be coupled with strategies to decrease IFP to avoid NP pumping out of the tumor through a high-pressure gradient from inside to outside the tumor. Thus, crossing/destroying the ECM and alleviating IFP contribute to NP homogenous diffusion and deep penetration [44,121]. The ECM can be degraded physically or enzymatically and stromal cells can be depleted or reprogrammed to improve NP penetration deep into the hypoxic core of solid tumors. Wenfeng et al. designed a dual-response shape transformation and charge reversal strategy with chemo-photodynamic therapy to improve the blood circulation time, tumor penetration, and retention, which finally enhanced the anti-tumor effect [122]. Rui et al. developed novel transformable nanomaterials designed to utilize the EPR effect more effectively. By tandem conjugation of the hydrophobic head (chlorin e6 (Ce6) or bilirubin (BR)), peptide to form hydrogen bond (Phe-Phe-Val-Leu-Lys (FFVLK)), and hydrophilic tail PEG, chimeric molecules that can form micelles (Ce6/BR-FFVLK-PEG) in aqueous solution are synthesized. Notably, the spherical micelles retain shape transformability. Evaluations at both cell level and animal level reveal that PTX2-TK@Ce6/BR-FFVLK-PEG exhibits superior biocompatibility and biodistribution, and suppresses 82.6% of in vitro cell growth and 61.8% of in vivo tumor growth at a common dose of intravenous injection, becoming a novel nanomedicine with extraordinary potential in cancer therapy [123].

#### 2.3.1 Nanosweeper: ECM degradation

The main component of dense ECM found in fibrotic (desmoplastic) PDAC tumors is high collagen content, reaching 12.8 ​± ​2.3% vol, compared to 1.4 ​± ​0.4% in the pancreas of healthy mice. Systemic injection of collagozome (100 ​nm liposomes loaded with collagen) reduced fibrotic levels by 5.6 ​± ​0.8% by only ∼1% of the injected dose reaching the pancreas over 8 ​h. Pretreatment with collagozome was shown to increase uptake of PTX micelles by 17% and produce 87% tumor suppression in the absence of eliciting metastasis or activating CTCs [45]. To overcome dense ECM, exosomes that act as a natural membrane were exploited for membrane protein delivery of native PH20 hyaluronidase. This formulation was used to enable DOX drug penetration and deep diffusion into tumor foci for enhanced tumor growth inhibition [124]. Another study reported the fabrication of a protein-free collagen nanosweeper composed of Au@silica nanorods coated with triphenylphosphonium bromide (TPP, mitochondria targeting cationic compound) and loaded with thermosensitive S-nitrosothiols capable of NO release upon heating and can also perform PTT upon NIR irradiation [125]. The liberated NO can activate MMP to break collagen fibers [46], which enhances cellular internalization by relaxing stiff ECM. Additionally, rapid interaction of superoxide anion with diffusible NO might produce the potent and very toxic reactive nitrogen species (RNS)- peroxynitrite (ONOO^−^), which can damage mitochondria by promoting nitrification and oxidative pressure, consequently leading to cell apoptosis [125].

As an additional strategy, pretreatment with free losartan (an angiotensin inhibitor) has been used for collagen I depletion to facilitate NP penetration. In one effort, losartan administration before injection of pH-sensitive cleavable liposomes (Cl-Lip) was shown to result in an enhanced antitumor effect. Collagen I reduction by losartan improved oxygen distribution in tumor tissues and allowed for deep percolation of Cl-Lip. Additionally, the tumor accumulation of NPs increased by 22.0%. The combined work of free losartan with PTX-Cl-Lip was more effective (59.8%) than PTX-Cl-Lip without losartan (37.8%) in a 4T1 breast cancer model [126] with significant (88.2%) anti-metastatic efficiency in mice [127].

ECM-degrading potential can be integrated into the NP structure. Superparamagnetic NPs linked to collagenase possess sufficient proteolytic enzyme stability, allowing the construct to drill through the ECM under magnetic fields in vitro [128]. Likewise, conjugation of bromelain (Br), as an enzymatic complex from the peptidase papain family possessing high proteolytic activity, to MSNs enabled the uptake of the particles in endothelial, macrophage, and cancer cell lines. Importantly, no significant effect on cellular viability was observed. In addition, Br-MSNs displayed an improved ability to digest and diffuse into the tumor ECM both in vivo and in vitro [129]. To translate a similar strategy to in vivo and for systemic use, one study employed the recombinant human hyaluronidase PH20 (rHuPH20) on the surface of PLGA-PEG NPs. Furthermore, grafting a low-density layer of PEG increased the serum half-life of rHuPH20 and quadrupled the accumulation and uniform tumor distribution of conventional DOX@PLGA-PEG NPs in 4T1 syngeneic mouse breast tumors [130]. Other strategies based on pulsed HIFU have also been employed to remodel the ECM. In one experiment, the aforementioned technique was utilized to enhance the ability of glycol chitosan nanoparticles (Cy5.5-CNPs) to target tumors with high hyaluronan and collagen contents in vivo. Injecting hyaluronidase or collagenase in an intratumoral manner, along with a low power of pulse HIFU, led to enhanced blood flow, reduced collagen contents, and improved CNP penetration. Furthermore, a 2.5-fold tumor targeting effectiveness in pulsed HIFU-treated mouse tissues was observed compared to the controls [131]. In another study, HAase treatment was used for vascular normalization to improve tumor oxygenation and the efficacy of PDT. This increased the tumor perfusion of nanomicelles conjugated with chlorine e6 (NM-Ce6) through an improved EPR effect by ∼2-fold, and can further enable metastasis targeting given the ability of HAase to effectively migrate from the primary tumor to its drainage sentinel lymph nodes (SLNs) [47].

#### 2.3.2 Stroma cell remodeling

**Fig. 11:**
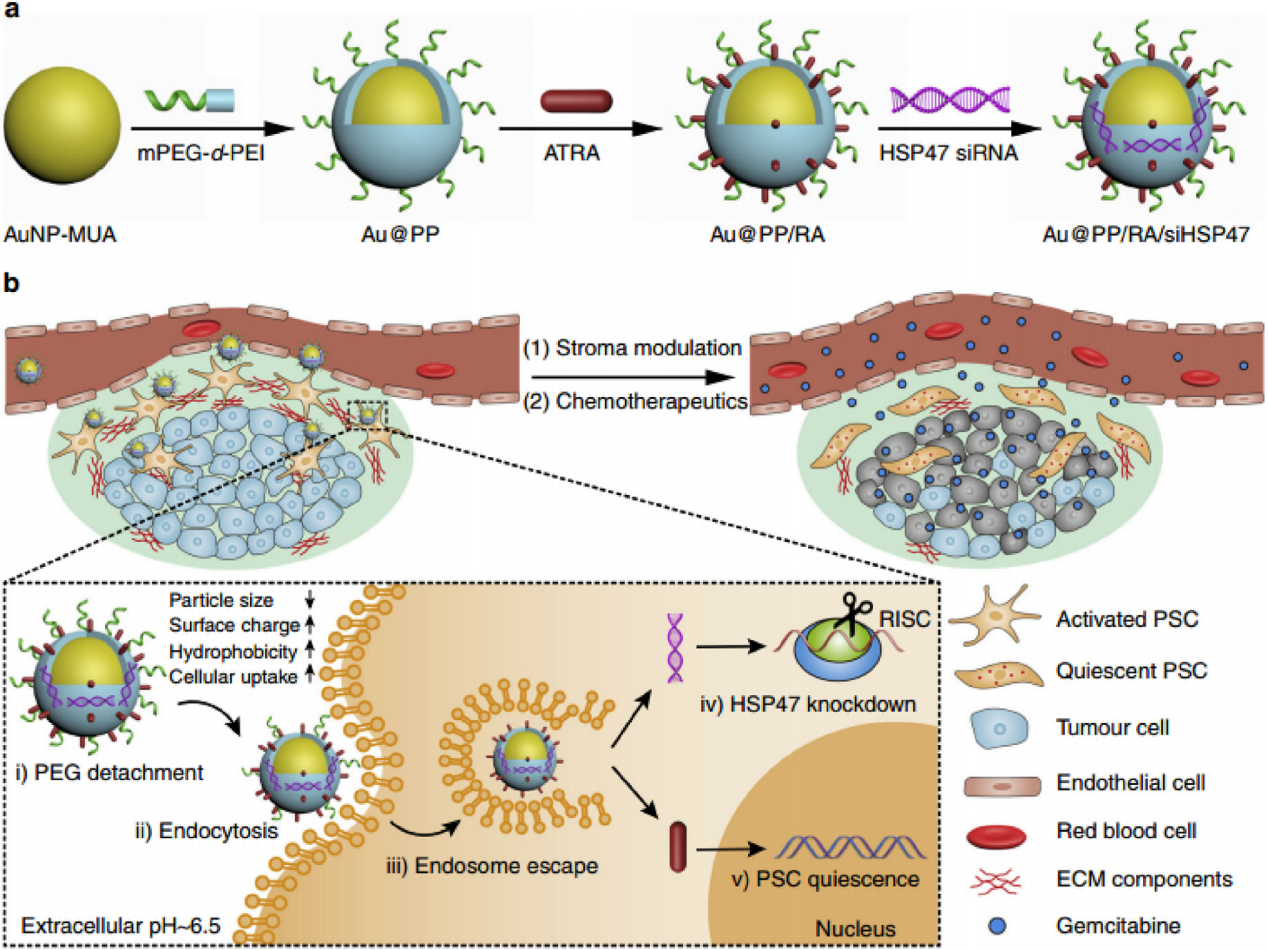
**Stroma cell reeducation strategy for ECM remodeling of desmoplastic tumors**. (a) Schematic of pH-responsive gold NPs-based system for co-delivery ATRA and HSP47 siRNA. Anionic ATRA and PEG-grafted polyethyleneimine (PEI)-coated gold nanoparticles electrostatically adsorbed siRNA. (b) Schematic of nanosystem stroma modulation and PSC re-education. In the acidic pancreatic tumor microenvironment (pHe 6.5), the nanosystem “activates” (PEG shedding, size decrease, charge rise, and hydrophobic ligand exposure) and exhibits pHe and ATRA dual-enhanced cellular uptake and HSP47 knockdown in PSCs. The desmoplastic stroma is homeostatically repaired, blood perfusion and medication delivery improve, and activated PSCs become quiet. Reprinted with permission [132]. Copyright 2018, Nature Publication Groups. (For interpretation of the references to colour in this figure legend, the reader is referred to the Web version of this article.)

Nonimmune cell reprogramming strategies are also possible to surmount desmoplasia cancers such as PDAC, which are characterized by a dense stroma and poor permeability and thus cannot take advantage of the EPR effect. One feasible formulation to tackle this problem is through the deactivation of pancreatic stellate cells (PSCs), which have been implicated in the pathogenesis of the tumor. In one particularly interesting study, PEGylated polyethylenimine (PP)-coated NPs of gold responsive against the TME were employed for the delivery of all-trans retinoic acid (ATRA), as an inducer of the quiescence of PSCs and enhanced adsorptive endocytosis, and siRNA targeting heat shock protein 47 (HSP47), as a molecular chaperone specific to collagen. In the acidic tumor microenvironment, the PEG coating of NPs is detached, which results in NP activation. More specifically, PEG shedding revealed hydrophobic ligands, reduced size (∼38 ​nm), increased charge (33.7 ​± ​0.7 ​mV), and promoted pHe (extracellular pH ∼6.5) and ATRA dual-enhanced cellular uptake. The construct had the ability to silence PSCs and inhibit ECM hyperplasia and thus promoted drug delivery to well-perfused pancreatic tumors. Although Au@PP/RA/siRNA resulted in less t_1/2,elim_ than Au@PP/siRNA at 9.04 ​h and 6.21 ​h, respectively, this formulation had a 2.5-fold greater accumulation in tumors than Au@PP/siRNA (Fig. 11) [132].

NPs often target fibroblasts in the TME, which restricts effective desmoplastic tumor treatment. However, such off-target features have inspired scientists to develop anticancer strategies hampering the expression of cytotoxic proteins secreted from tumor-associated fibroblasts (TAFs). For one, protamine DNA complexes coated with lipids were employed to encapsulate plasmids that encoded the secretable TNF-related factor sTRAIL. The construct was subsequently administered to murine models of human desmoplastic bladder carcinoma. As a result, apoptosis was triggered in tumor cell nests neighboring the TAFs. Moreover, sTRAIL was capable of reverting residual fibroblasts to a state of quiescence, which further compromised tumor growth and ECM remodeling [133].

Another formulation with the aim of reducing IFP to enhance NP penetration and simultaneously reduce pulmonary metastasis of breast 4T1 cancer has been reported. This NC involved a gelatin shell containing imatinib (tyrosine kinase inhibitor) and DTX and quercetin (PI3K/MMP9 inhibitor) loaded in the positively charged lipid core. MMP degradation of the gelatin shell upon entrance of NP into the tumor interstitium through EPR allowed the enzyme-controlled release of drugs from NPs. IFP reduction occurred due to the actions of imatinib, which blocked PDGFR-β and disrupted the interaction of TAFs with ECM, and anti-metastatic activity occurred due to the inhibition of MMP activity by quercetin [134].

#### 2.3.3 Micropropellers: tumortropic vectors

Endowing nanorobots with capabilities such as self-propulsion and rotation improves the efficiency of a medibot, enabling it not only to move toward its target but also to drill into the barriers on its way (More studies were reviewed in Ref. [135]). Acting as biological motors possessing inherent motility properties, various microorganisms (bacteria) as well as specific cell types (sperm, macrophages) can be used as natural NCs for the design of bioinspired robots and for drug delivery purposes [48]. Additionally, synthetic motors are powered by external sources, such as light, US, glucose [136,137], or magnetic fields, enabling NP convection through ECM [138]. First, the magnetically guided motion of nanobots is designed to automatically move through high velocities and in human serum. Nanobots were fabricated by chemical conjugation of magnetic Fe_3_O_4_ NPs and an anti-epithelial cell adhesion molecule antibody (anti-EpCAM mAb) by a GSH linker to DOX-laden multi-walled carbon nanotubes (CNTs), where Fe_3_O_4_ NPs acted as a cap for the on-demand release of DOX. This format was based on magnetic Fe_3_O_4_ NPs, as they impart autonomous propulsion ability by a self-oxygen bubble generation ability from tumor-enriched H_2_O_2_ to relieve tumor hypoxia for deep penetration of nanobots. Additionally, the nanobots preferably release DOX in the acidic lysosomal compartment due to reduction of the amide linkage, which opens the Fe_3_O_4_ gate for DOX release. Thus, self-assisted nanobots tow drugs into ‘far-reaching’ sites in the depth of tumors [139].

**Fig. 12:**
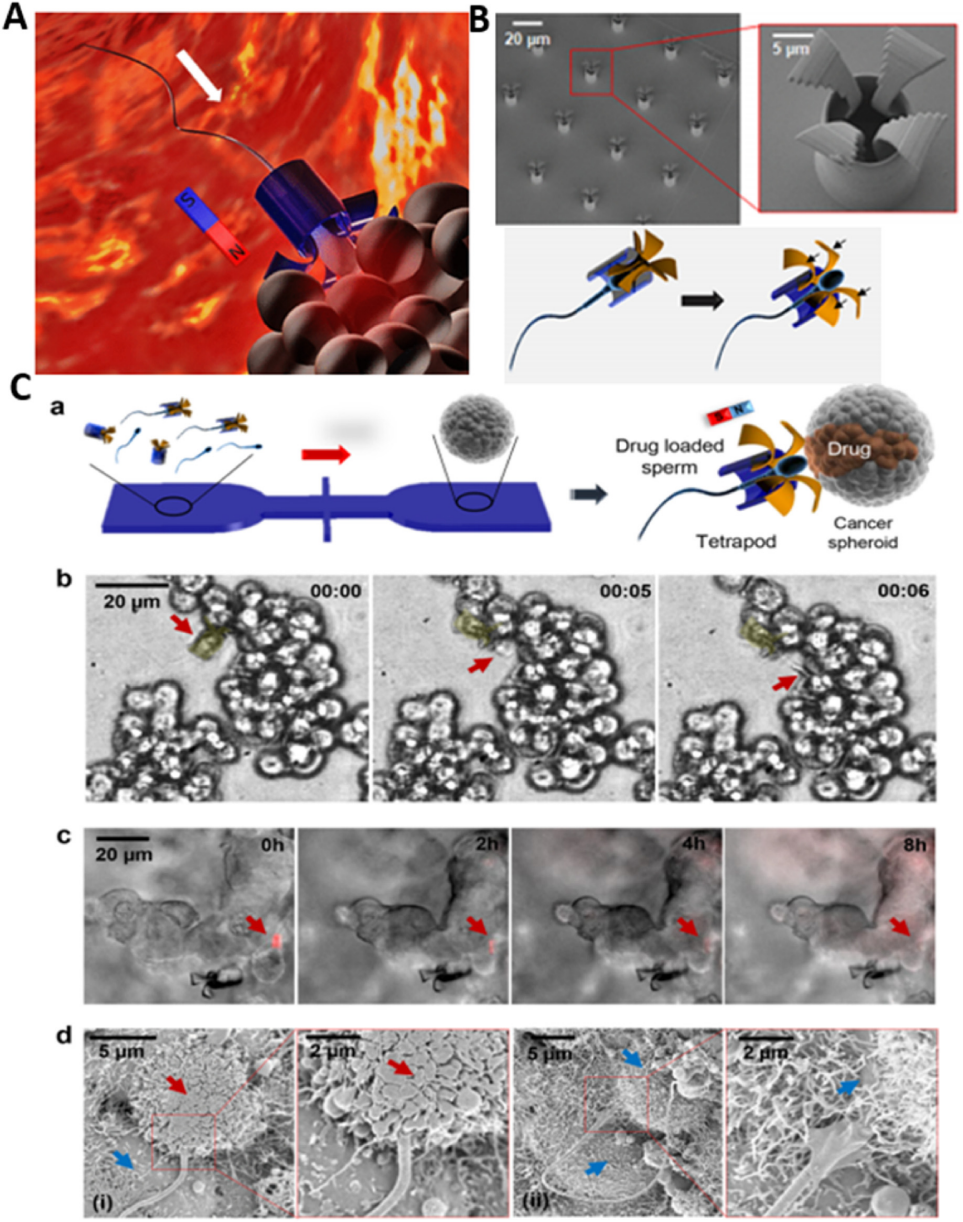
**Sperm-bioinspired microswimmer for tumor cell internalization** (A) design concept. (B) SEM images of 3D-printed tetrapod microstructures. (C) (a) Illustrations of the working principle for the release of drug-loaded sperm on a microfluidic chip. (b) Sperm liberation process upon hitting arms with HeLa spheroids. (c) Distribution of DOX-HCl in HeLa cells. (d) SEM images showing HeLa cell fusion with (i) DOX-HCl-loaded sperm or (ii) unloaded sperm. Live cells are shown with blue arrows, while red arrows indicate apoptotic cells. Reprinted with permission [141]. Copyright 2018, American Chemical Society. (For interpretation of the references to colour in this figure legend, the reader is referred to the Web version of this article.)

In one development, a fuel-free NIR light-powered Janus mesoporous silica nanomotor was fabricated that exploits the macrophage cell membrane (MPCM), not only avoiding biofouling in biological media but also freely and actively searching for cancer cells. This system can inject guest molecules into cancer cells by the NIR-activating photothermal effects of the Au half-shell, allowing nanomotors to thermomechanically perforate the cytomembrane for guest molecule injection. This approach integrates the functions of active seeking, cytomembrane perforation, and thermomechanical therapy in nanomotors, which may pave the way for the application of self-propelled motors in biomedical fields [140]. Magnetic-guided natural micromotors are also adopted as biocompatible carriers with a high drug-loading capacity and the capability to internalize into target cells. For example, a sperm-hybrid micromotor has been reported that consists of a motile sperm cell-laden DOX with four arms (tetrapods) that holds sperm and can only release it when the arms are mechanically bent by sperm-cell membrane close contact. More specifically, sperm liberation is allowed when the bio-hybrid micromotor hits and fuses with the tumor cell membrane, followed by mechanically-triggered drug release, as validated in 3D tumor spheroids (Fig. 12) [141]. Self-enriched with a chain of magnetic iron-oxide nanocrystals, the magneto-aerotactic migration behavior of MC-1 bacteria toward a low oxygen gradient under a magnetic field has been described for the delivery of 70 types of drug-laden nano-liposomes, with up to 55% penetration of MC-1 ​cells into hypoxic HCT116 colorectal xenografts [49]. Recently, drug-internalized bacterial swimmers have been used for the precise delivery of DOX without the involvement of NPs. DOX was loaded into live MSR-1 ​cells by supplementing the medium with iron ions, facilitating DOX internalization [138].

#### 2.3.4 Cluster bombs: size/charge changing nanoparticle

A recently appreciated Trojan system resembles a ‘cluster bomb’ or ‘jet-loaded bombs’ in that they are assembled through reversible crosslinking of thousands to millions of small-sized particles that form a single larger-sized particle. The larger size (>100 ​nm) allows selective and specific accumulation of nanobombs in tumors using the EPR effect. Later, to enable NP diffusion throughout the tumor region, size/charge-changing capability is activated to decompose nanobombs into small (positively) charged particles with high penetration to drill the ECM. Nanobombs differ based on stimuli responsiveness and can switch their charge/size to internal stimuli (pH [142,143], oxygen, redox [144], and tumor-specific proteases (e.g., MMPs [145], hyaluronidase [146]), external stimuli (light, US, and magnetic fields) or combinations of both. Regarding the effect of NP size, ultra-small particles are desirable, as gold NPs (Au@tiopronin) could be localized to and penetrate deeply into breast cancer cells, multicellular tumor spheroids, and tumors in vivo. NPs with sizes of 2 ​nm and 6 ​nm have been shown to highly accumulate in the cytoplasm and nucleus of tumor cells in vivo, compared to 15 ​nm NPs, which aggregate only in the cytoplasm [147].

**Fig. 13:**
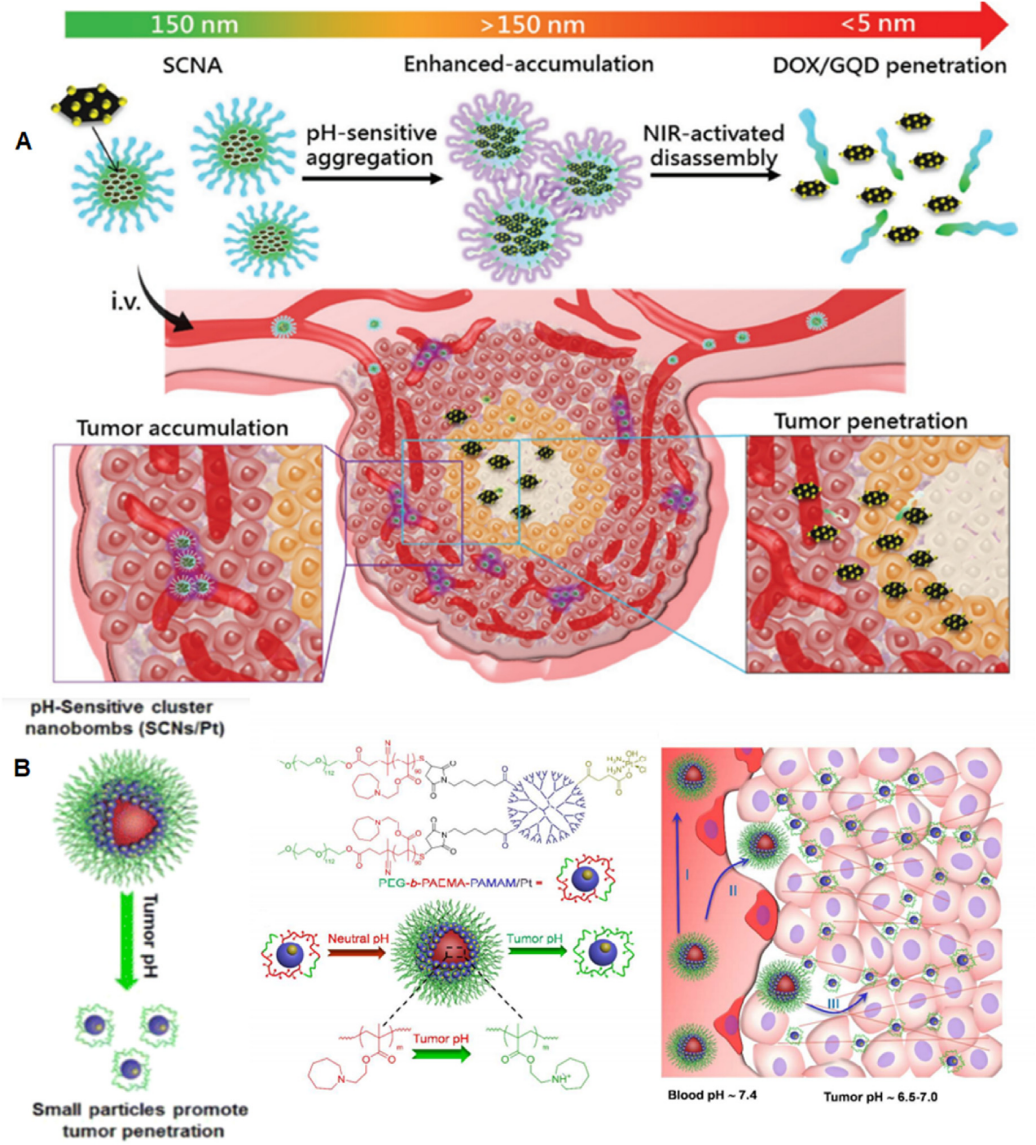
**Schematics of nanobombs for crossing the ECM barrier.** (A) Example of light-triggered decomposition of pH-assembled nanobombs in tumors. Reprinted with permission [143]. Copyright 2017, John Wiley and Sons, Inc. (B) Dendrimer-based cluster nanobombs with instantaneous size switching in a tumor acidic environment enable improved tumor penetration. Reprinted with permission [148]. Copyright 2016, National Academy of Science.

A size-changeable graphene quantum dot (GQD) nano-aircraft (SCNA) is a light-responsive nano bomb that can penetrate tumors and deliver anticancer drugs. The nano-aircraft is composed of pH-sensitive polymer-functionalized GQDs less than 5 ​nm. These GQDs aggregate in the weakly acidic tumor environment but are stable and stealthy at physiological pH. NIR irradiation disassembles 150 ​nm of SCNA into 5 ​nm of DOX/GQD at the tumor site, allowing deeper penetration into the tumor tissue. DOX/GQD that enters the tumor can kill nearby cancer cells. This SCNA suppressed xenograft tumors in 18 days without distal harm with combinational therapy. This sophisticated strategy delivers hierarchically targeted and penetrated drugs and energy to deep tumors because DOX and GQD fluorescence signals are visible throughout the entire tumor region and foci (Fig. 13A) [143]. Another example where size alteration of the nanoparticle facilitated its penetration into deep tumor regions includes a polymeric clustered NP (iCluster) with a size of ∼100 ​nm. However, when accumulated in tumor sites, the lower pH of the TME triggered the release of platinum prodrug-conjugated poly (amidoamine) (PAMAM) dendrimers (diameter ∼5 ​nm), which enabled both cellular internalization and tumor penetration. The platform was verified against various tumors, such as metastatic cancer, drug-resistant cancer, and poorly permeable pancreatic cancer, in vivo (Fig. 13B) [148]**.**

Another work described a size-changeable polymer micelle system (MPEG-PLA-ss-PEI-DMMA) with a dual shell that enlarges in acidic pH and shrinks in intracellular glutathione (GSH). It treated drug-resistant breast cancer by directly delivering anticancer drugs to the nucleus of multidrug-resistant (MDR) tumor cells, unlike micelles without size-changing capacity, which tend to collect NPs in the cytoplasm of MCF-7/ADR cells in vivo [149]. MSN-based worm-like NPs capable of size changing from 50 ​nm to 5 ​nm have also been fabricated. DOX-loaded MSNs (∼50 ​nm) were capped with tungsten disulfide quantum dots (WS_2_ QDs ​= ​5 ​nm), which were themselves modified with tLyP-1 peptide (WS_2_-HP) as a tumor homing peptide, together forming DOX@MSN-amide-WS2-HP, a “Cluster Bomb” to target deep-seated tumor cells. The benzoic-imine bond linkers between the “dispenser” and the “bomblets” were stable under normal pH but labile at pH 6.8, which made the platform break into two parts following its arrival at the TME. The DOX@MSN-NH_2_ part was capable of targeting tumor cells, and WS_2_-HP could penetrate deep tumor regions by NIR-triggered PTT. Therefore, the platform was capable of exerting excellent antitumor effects both on the surface and in the deep regions of the tumors [150].

In another study, the complementary strand of the triplex-forming oligonucleotide sequence mediates ultra-small gold nanoparticle self-assembly. The sunflower-shaped, 200 ​nm nanostructures have high photothermal and NIR absorption. The enormous nanostructure broke apart and created 2-nm Au-POY2T NPs containing the c-myc oncogene silencing sequence that could directly target MCF-7 ​cell nuclei after NIR light exposure. This formulation resulted in a 1.7-fold increase in the uptake of 2 ​nm particles resulting from the degradation of nanoclusters compared to single Au-POY2T NPs at 24 ​h posttreatment. Similarly, coupled with NIR illumination after a 12-h preincubation time, there was a 170% increase in the accumulation of NPs in the nucleus compared to the control [151].

**Fig. 14:**
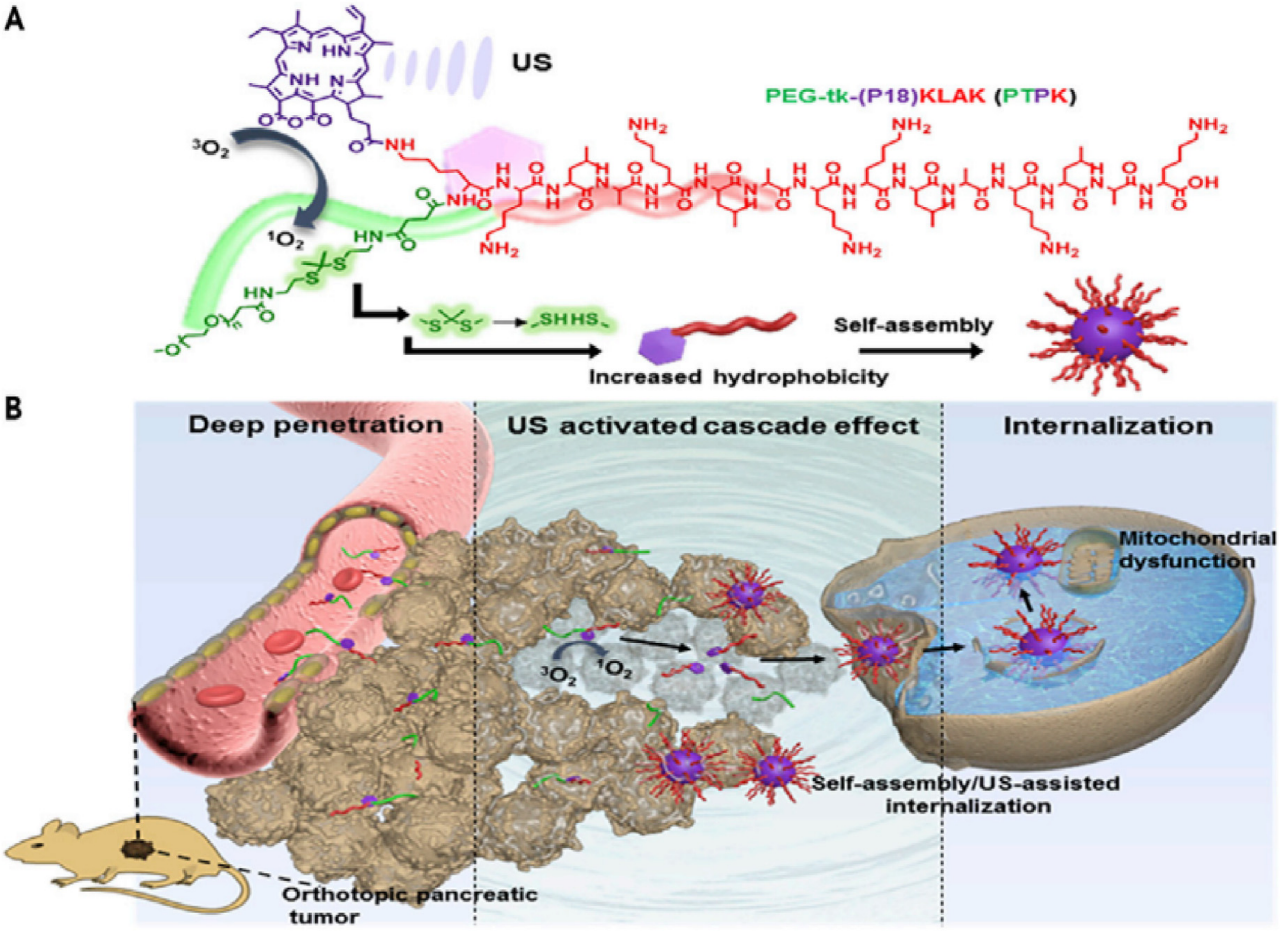
Orthotopic Pancreatic Cancer Treatment with Polymer-Peptide Conjugates: Ultrasound-Activated Cascade Effect (A) PTPK molecular structure and US activation cascade. (B) Synergistic PTPK internalization and US-induced cascade impact limit tumor development. Reprinted with permission [152]. Copyright 2020, Elsevier.

Finally, the US-actuated cascade process for singlet oxygen generation has been attempted by a polymer-peptide conjugate PTPK (PEG-thioketal (tk)-(P18) KLAK), which is capable of deep tissue penetration. US mediates ^1^O_2_ generation from the sonosensitizer purpurin 18(P18). Next, ^1^O_2_ cleaves the thioketal bond to detach PEG chains, promoting the in situ self-assembly of PK NPs with considerable cellular internalization. Unlike the studies discussed above, here the generation of larger NPs (∼38 ​nm) from a small-sized, single-stranded PTPK (∼7 ​nm) was adopted. US was applied in two ways to benefit NP penetration by acting as an external stimulus to enhance EPR and a cascade reaction to promote NP generation by cleaving the SS linkage to favor ‘sonochemical internalization’. The intensity of fluorescence signals of PTPK-treated mice was ∼1.6- and 3.6-fold higher than those in the PK and PPK groups, respectively. Additionally, the PTPK penetration distance was ∼153.2 ​± ​28.7 ​μm, almost 3-fold deeper than that of PK NPs (48.3 ​± ​17.1 ​μm) (Fig. 14) [152].

### 2.4 Engineering of the nanoparticles for intracellular accumulation

The last barrier to NP drug delivery for cancer therapy is the challenges to cross the tumor membrane, enter the nucleoplasm and release the drug. Strategies to cross this barrier can be classified into NP design, receptor-based, and receptor-free approaches. Also, various strategies for organelles targeting can be found in reference [50].

#### 2.4.1 Properties and optimization of nanoparticles

The physicochemical [51] (e.g. size, shape, contact surface area, the adhesive force between NPs and the cells/matrix, composition, and surface chemistry) and mechanical properties [153] (e.g. elasticity and stiffness) of NPs determine the penetration specificity, rate, depth and pathway of NPs into solid tumors. With regard to the effect of NP shape, antibody (trastuzumab)-coated rod-shaped NPs have been shown to impart superior cellular binding and internalization than sphere NPs and nanodiscs in vitro, which pinpoints the role of NP morphology in antibody specificity and avidity [154]; however, spherical NPs may also result in enhanced cellular uptake rates over rod- and disc-shaped particles. This discrepancy arises from other factors, including NP composition, material, chemistry, analytical tools, and inappropriate cellular models [155]. With regard to NP shape and the effect of elasticity on the preservation of NP structure during cell penetration, stiff silica nanocapsules (SNCs) can preserve the spherical shape of NPs, which favors their cellular uptake. However, soft SNCs elicit reduced cellular binding and endocytosis rates because they become deformed due to specific ligand–receptor contacts and membrane wrapping [156]. NP elasticity and transformability direct tumor accumulation, as shown in an orthotopic breast tumor model, with marked accumulation of soft nanolipogels (NLGs) in the tumor and preferential uptake of elastic NLGs in the liver [157].

When considering the effect of cellular models, the effect of NP shape on tumor internalization varies in 2-dimensional (2D) monolayers versus 3-dimensional (3D) spheroids. Elongated polystyrene (PS) NPs with an identical chemical composition, fixed volume, and equal zeta potential (uniform coating of NPs with poly propylene imine dendrimer G4) but with different aspect ratios (ARs) of Z1 (AR ​= ​1), Z2 (AR ​= ​2.8) and Z3 (AR ​= ​7.5) have been generated. The results showed that the elongated PS particles have an optimal AR in the 3D spheroids, with the deepest penetration into the foci of solid tumors in the order of Z2 (103 ​μm) ​< ​Z1 (76 ​μm) <Z3 (70 ​μm). In contrast, increasing AR (Z3) in 2D cell monolayer culture was associated with the decreased uptake of NPs. This was consistent with the uptake mechanism of NPs, as the intercellular pathway was the main route for Z2 NPs because their fluorescent signal was more affected (i.e., faded) by verapamil treatment (P-gp efflux protein) compared to Z1 and Z3. Additionally, the penetration pathways of NPs in tumor spheroids involve both intracellular and intercellular penetration pathways, which suggests the superiority of 3D systems as reliable models over 2D systems for the study of NP behavior [51]. With regard to the surface charge, positively charged particles such as PAMAM dendrimers, have been shown to result in rapid penetration of cellular membranes through adsorptive endocytosis [158]. Additionally, small particles can more easily penetrate through the membrane than larger particles [159]. Furthermore, active targeting of cationic dendritic polyglycerols (dPGs) decorated with anti-EGFR single-domain antibodies can produce ultra-small NPs with high drug accumulation and retention and facilitate intracellular uptake of NPs through specific interactions, such as receptor-mediated endocytosis [53].

“DART” NPs have been fabricated that display minimum nonspecific adhesivity to ECM while promoting specific receptor-mediated targeting of primary as well as metastatic breast solid tumors (GBM) in vitro, ex vivo, and in vivo. DART is an optimized polymeric NP composed of PLGA-PEG-ITEM4 (∼95 ​nm), where ITEM4 is an antibody targeting the extracellular domain of fibroblast growth factor–inducible 14 (Fn14). It was found that NP formulation with 10% PEG and 1% ITEM4 density retained a cell surface receptor-specific binding affinity for tumor cell–specific uptake, an improved blood circulation time, and possible biodistribution. Flow cytometry analysis showed a ∼2.5-fold increase in the binding of PLGA-PEG-ITEM4 NPs to 231-Luc cells compared with PLGA-PEG NPs. After systemic injection, PLGA-PEG-ITEM4 NPs accumulated ∼2-fold more than the nontargeted PLGA-PEG-IgG particles in 231-Luc tumors. PTX-PLGA-PEG-ITEM4 out-performed Abraxane, an FDA-approved nanodrug formulation for PTX delivery, in a primary triple-negative breast cancer model and an intracranial metastatic model [160].

It is well recognized that the spatial presentation of the ligand, which is governed by valency, affects the targeting ability of multivalent ligand-modified NPs. Because receptor overexpression varies between cancer kinds and stages, NP must be optimized for ligand valency based on tumor receptor distribution. Accordingly, multivalent magnetic NPs (MMNs) of various valences were coated with varied numbers of the multivalent dendritic polyethyleneimine ligand cluster PRn (n ​= ​2, 4, and 8), which was altered by raltitrexed (a ligand for the folate receptor). Fe-PR4 was the best valency for treating high FR overexpressing KB cells with decentralized receptor distribution since Fe-PR8 can induce steric hindrance in the confined binding area and Fe-PR2 was negative in statistical rebinding. Instead, the extra ligands on Fe-PR8 would improve statistical rebinding in vitro and in vivo in HeLa cells with moderate FR overexpression and clustered receptor display [161].

Without NP design, cancer cells can selectively ingest NPs. In an orthotopic mouse model of human glioma and human tumor xenografts in mice, carbon QDs functionalized with several amino groups, and linked-carboxyl may systemically bind to the elevated neutral amino acid transporter 1 with great affinity. These NPs resemble large amino acids and stacking interactions to load aromatic drugs for cancer theranostics. After 1 ​h, HeLa cell membranes showed LAAM TC-CQDs entering the cytoplasm. After 6 ​h, several NPs penetrated the nuclei. By 8 ​h, the nucleus had more NPs than the cytoplasm [162].

**Fig. 15:**
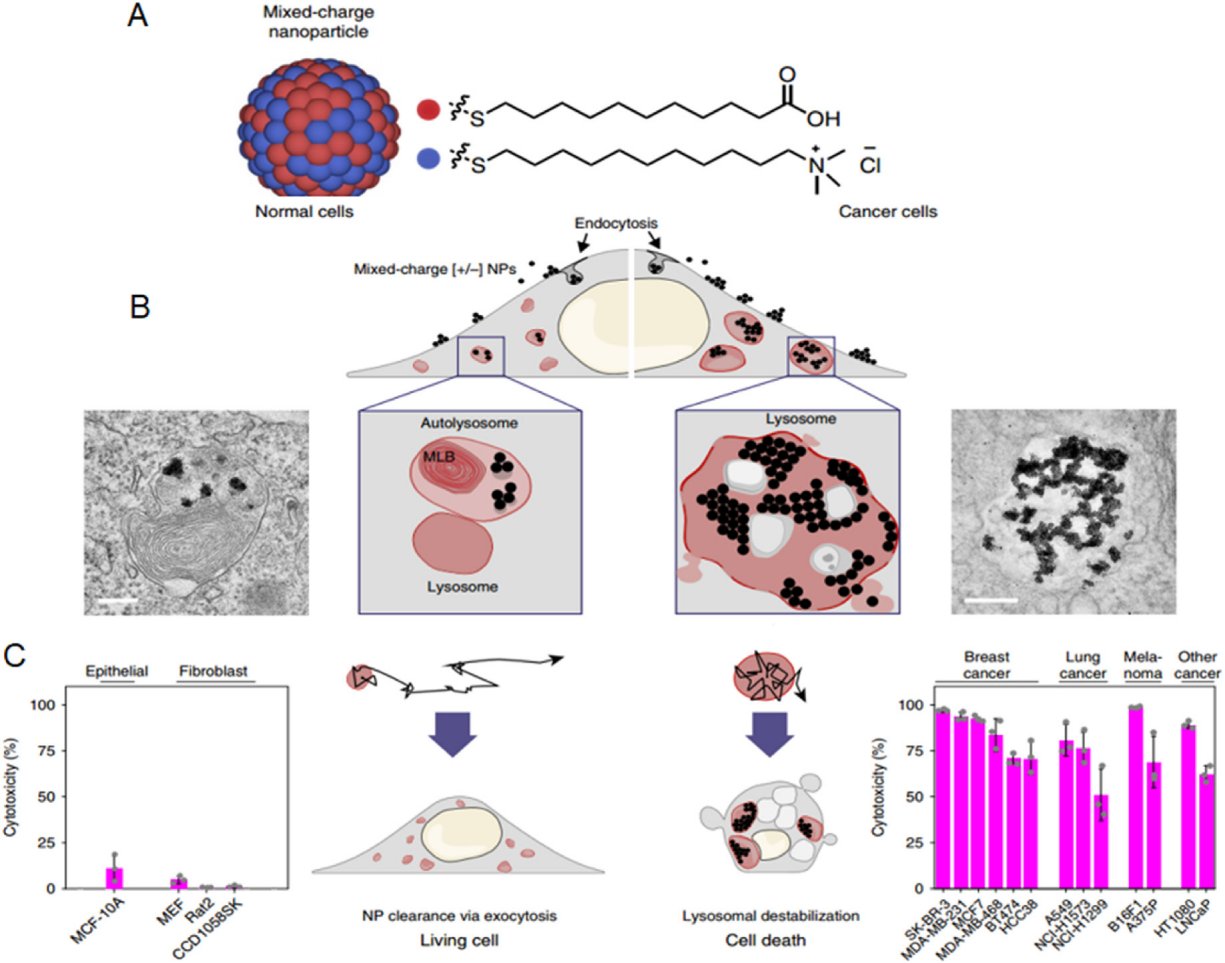
**NP surface charge for selective targeting of cancer cell organelle.** (A) Positively charged (blue) and negatively charged (red) ligands make up the NP design. (B) The idea behind and the method by which mixed-charge NPs crystallize in cancer lysosomes and destroy cancer cells in a targeted manner. Effectiveness against various cancer cells. Reprinted with permission [163]. Copyright 2020, Nature Publication Groups. (For interpretation of the references to colour in this figure legend, the reader is referred to the Web version of this article.)

NP optimization has been used to target cancer cell lysosomes and disrupt the tumor cell membrane barrier. This study showed that controlled crystallization of mixed-charge NPs covered with 20:80 ratios of positively and negatively charged ligands could selectively target lysosomes to escape caspase-dependent apoptosis and selective cancer cell death. Structure-wise, monolayers self-assembled from anionic 11-mercaptoundecanoic acid (MUA) ligands and cationic N, N, N-trimethyl (MUA) ammonium chloride (TMA). MUA ligands are protonated and neutral at low pH but mostly deprotonated and negatively charged at pH 7.4, while TMA ligands are always positive. This NP formulation targets cancer cells (organelles) by forming small, endocytosis-prone (50–100 ​nm) NP clusters at cell surfaces and giant, well-ordered nanoparticle assemblies and crystals of ∼2 ​μm inside cancer lysosomes. These assemblies prevent NP exocytosis (20% in cancer cells vs. 50% in normal cells after 6 ​h) and cause lysosome swelling, which gradually compromises lysosomal membrane integrity, impairing lysosomal functions and ultimately causing selective cell death in 13 cancer cell lines without toxicity to normal fibroblast or epithelial cell lines. The electrostatic model predicted that NP accumulation up to 27% vol/vol for HT1080 and 45% for MDA-MB-231 versus 74% to full packing was sufficient for lysosome expansion but not imminent rupturing (Fig. 15) [163]. A similar principle, exploiting intratumoral formation of gold NPs inside cells for successful photothermal ablation of tumors in vivo has been attempted using PEG as a carrier for positively charged Au^+3^ ions forming Au-PEG clusters. Furthermore, progressive intracellular bio-mineralization of Au-PEG clusters can occur en route starting from the cell membrane by extracellular proteins, membrane proteins, and finally terminal reduction upon entry into the nucleus to produce plasmonic gold NPs inside MCF-7 ​cells by producing plasmonic gold nanoparticles at micromolar concentrations within 30 ​min of application [52].

#### 2.4.2 Oncotropic vector: receptor-mediated transport

Although the internalization rate is important in the sense of anticancer drug targeting, finding the tumor cell for on-target effects in a large pool of other off-target cells (e.g. normal cells, stroma cells) in order to avoid toxicity and minimize drug dosing is essential. Tumor-specific targeting moieties are chosen with minimum nonspecific binding to cellular, intravascular, and extracellular components [160]. To increase the chance for precise NP targeting of on-target cells, receptor-mediated targeting is further shifted into sophisticated formats involving receptor design, multifunctional, lock and key (logic-operated) gates and artificial receptors.

##### 2.4.2.1 Logic gate

**Fig. 16:**
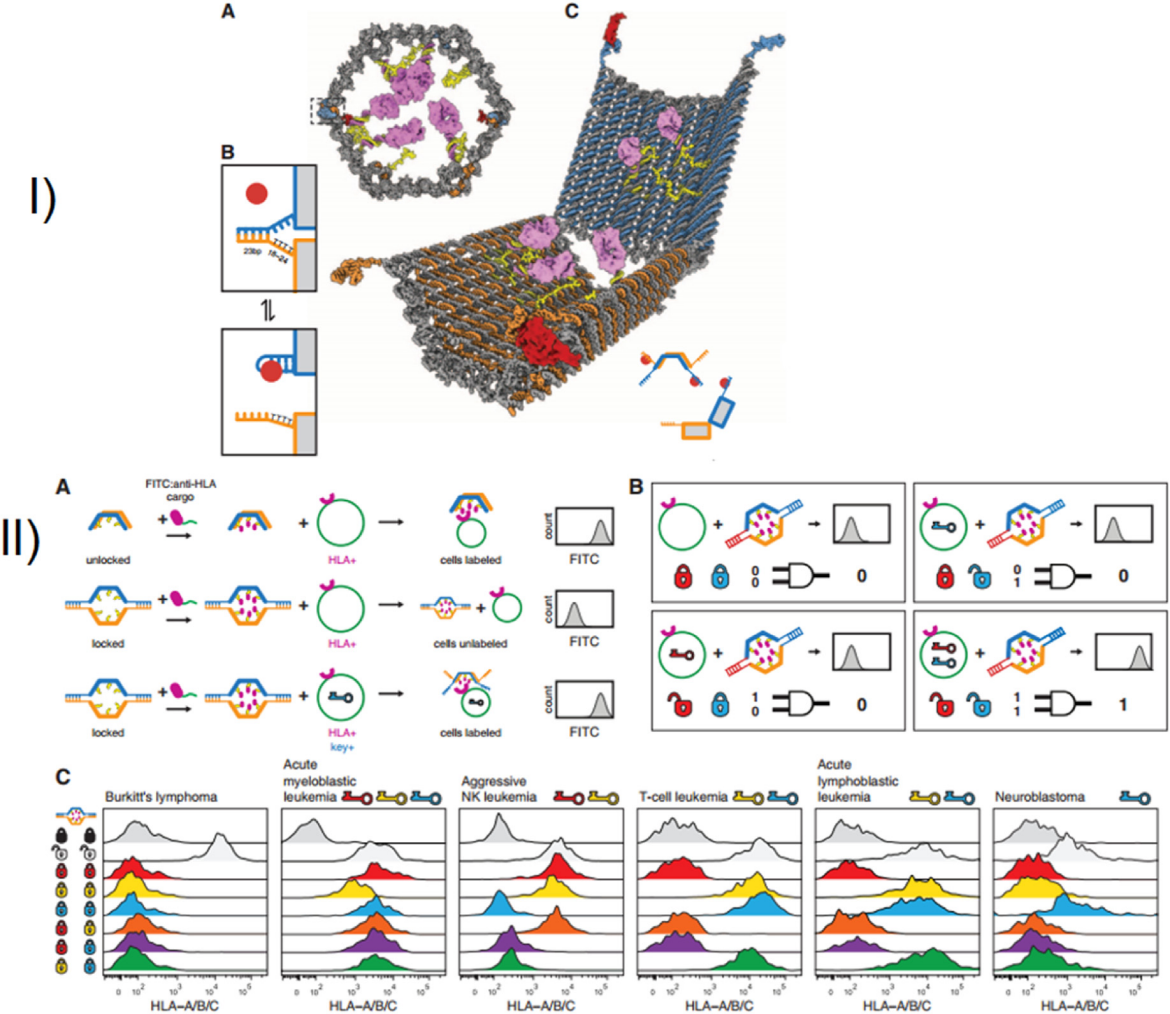
**Logic gate activation of a DNA nanorobot (oncotropic vector) allows selective recognition of different types of tumors. I).** Front orthographic schematic of a protein-carrying closed nanorobot (A). Left (boxed) and right DNA-aptamer locks safeguard the device's front. The aptamer lock mechanism is a blue DNA aptamer and a partially complementary strand (orange). Lock antigen keys can keep it separated (red). The lock duplex is always 24 bp, while the non-aptamer strand has an 18–24 base thymine spacer. (C) A protein-released nanorobot. Back scaffold hinges separate blue and orange domains. II) Logic-gated nanorobot activation of different cell types. Nanorobots carry fluorescently tagged HLA A/B/C-specific antibody Fab’ fragments. Nanorobots bind to unlocked HLA-A/B/C-expressing cells (top). Key cells (middle) keep them quiescent, but key ​+ ​cells activate them (bottom). Nanorobot activation truth table. Cell-expressed chemical keys activate the AND gates in aptamer-encoded locks. Colors like a lock and key. Nanorobot conformation is output from aptamer-antigen activation. (C) Eight nanorobots (10–100 ​fmol, loaded with anti-HLA-A/B/C antibody fragments at a molar excess of 20) were tested with six cell types expressing various antigen key combinations for 5 ​h. Each histogram displays cell number versus fluorescence from anti-HLA-A/B/C tagging. Reprinted with permission [165]. Copyright 2012, American Association for the Advancement of Science. (For interpretation of the references to colour in this figure legend, the reader is referred to the Web version of this article.)

The same concept of using ‘AND, OR, NOT’ as Boolean logic operators, which allows one to narrow search results to increase specificity, is utilized in the design of dynamic DNA nanostructures called ‘DNA nanorobots’. These gadgets are capable of performing several tasks from transporting molecular payloads to cells to sensing cell surface inputs (antigens ​= ​keys), which induce reversible shape changes (e.g., flattening a tubular NC) to expose a concealed drug payload for therapeutic performance (e.g., inducing tumor vascular infarction) [100]. This system employs ‘aptamer-encoded logic gates’ and utilizes toe-hold mediated strand displacement reactions to open a ‘lock’ by ‘key oligonucleotide strands’. This means that it is only the right combination of keys (e.g., tumor-specific surface antigens) that can open the aptamer lock placed on the exterior surface of a closed nanostructure containing drug payloads [164]. Along this line, a nanorobot has been formulated that contained various payloads, such as antibody Fab' fragments and Au NPs. Two AND logic gate DNA-aptamer locks were placed on the front of the barrel-shaped device to allow opening and activation of the nanorobot only in the presence of two different protein antigens. By using different lock combinations of sgc8c, 41t, TE17 aptamers, and fluorescently labeled antibodies against human HLA-A/B/C Fab', this system was capable of reliable differentiation of six different cancer types, supporting application in tumor-specific targeting and drug delivery (Fig. 16) [165]. More examples of this concept can be found in our previous work [164].

##### 2.4.2.2 Dual targeting

Another way to encourage tumor-specific selectivity is by using multifunctional NPs that work through cooperativity in binding to more than one (usually dual targeting) overexpressed cancer cell surface receptor to overcome the issue of low-level expression of the same markers by off-target cells, which results in nonspecific NP delivery and toxicity [166]. Using a dual-receptor targeted technique, peptide-targeted liposomes target multiple myeloma's overexpressed VLA-4 and LPAM-1. The NP design was comprehensively tuned for the valency of each targeting peptide at 0.75% VLA4pep and 1% LPAM1pep to increase cellular uptake above single targeted liposomes. This formulation had a cooperative ratio of 4.3 and increased uptake for myeloma cells that co-express both VLA-4 and LPAM-1 receptors but not for cells that express only one or neither, increasing myeloma targeting selectivity by 28-fold [167].

Another format of dual targeting can be achieved by using moieties that can target several types of cells at once. For example, Arg-Gly-Asp peptide (RGD) can target α_v_β_3_ integrin receptors, which are expressed by both cancer cells and TECs. Thus, it can enhance nanodrug accumulation and penetration using RGD as a homing peptide. In one preparation, dual-targeted synergistic RGD-coated polymer-lipid hybrid NPs loaded with DOX and mitomycin C (RGD-DMPLN) were prepared for the treatment of TNBC lung metastases. A moderate RGD density on NPs showed the highest accumulation in lung metastases by targeting both tumor vasculature and cancer cells, resulting in a 31-fold vs. 4.7-fold reduction in the burden of lung metastases and a 57% vs. 35% longer median survival time compared to control (nontargeted NPs) [168]. Metal-organic framework (MOF) NPs target TAMs and cancer cells using a maltotriose moiety, a chain of three glucose molecules. Maltotriose-PCN-224-0.1Mn/0.9Zn targets TNBC tumor cells and TAMs via the glucose transporter and CD206 (mannose receptor-1) receptors when exposed to LED light [169]. Various formats of dual targeting strategies can be found in our previous work [166].

##### 2.4.2.3 Artificial receptor (AR)

For some tumors, especially drug-resistant ones such as TNBC, tumor cell heterogeneity results in a limited number of cancer biomarker receptors available for active targeting. This can be surmounted by the formation of artificial receptors (ARs). ARs are mostly attempted using metabolic engineering, which allows the incorporation of unnatural sugars through a biorthogonal click reaction. However, other simple methods have also been introduced. For example, the insertion of biotin as an AR is recognized by pHLIP, a low-pH plasma membrane insertion peptide (38-aa). After insertion into the lipid bilayer, the extracellular tumor acidic microenvironment utilizes the C-terminal tail translocation of pHLIP to anchor cells through the α-helix and thus exposes N-terminally conjugated biotin on the cell surface. Biotin-labeled cells, including cancer cell spheroids, cancer cells, and an in vivo tumor, can actively sense and couple Cy5-streptavidin conjugates for cancer theranostics [170]. More examples of ARs can be found in Ref. [166].

#### 2.4.3 Receptor-free uptake

Another strategy that involves receptor-free uptake of nanoparticles can be envisioned by the natural tropism of biomimetic and synthetic micropropellers and Trojan systems. While micropropellers coupled with external actuators such as magnetic fields allow for the mechanical opening of a cell membrane [171], live cells such as bacteria act as Trojan horses for NP delivery and deep tumor internalization [172]. Exosomes are another beneficial tool that allows for NP delivery to cancer cells. For example, exocytosis of endocytosed DOX-loaded PSiNPs from tumor cells produces exosome-coated DOX@E-PSiNPs, which increase blood vessel extravasation, tumor accumulation, deep tumor penetration, and cellular absorption in bulk cancer cells and cancer stem cells (CSCs) [173].

## 3 Conclusion and future perspective

Specific characteristics of tumor cells, such as rapid growth, genomic instability and population dynamics, can cause a number of changes in tumor cells and their surroundings. These changes in the surrounding environment are due to the high need of tumor tissue for oxygen and nutrients and their high metabolism [174]. The changes in more detail include tumor tissue acidity and hypoxia, as well as an abnormal angiogenesis system and EPR. Even the degree of genome instability causes a set of neo-antigens or an increase in the expression of a particular receptor or biomarker in them. These unique features of tumor tissue compared to normal tissues inspired the design of NPs that have more efficacy in the tumor environment than in the normal tissue environment.

After the integration of nanomedicine as a viable option for cancer therapy, tumor features became more important than ever and gave rise to a great deal of hope as nanocarriers could be designed using chemistry based on the unique characteristics of tumors. The first and foremost tumor feature was the high gap between adjacent endothelial cells in the tumor vasculature ranging from 400 to 700 ​nm - something that assist them in fulfilling the high demands for oxygen and nutrients. Based on this simple principle, the NPs should be able to pass through tumor endothelial gaps but not the endothelial lining of normal tissues as they diplay tighter junctions between endothelial cells. Accordingly, the first EPR-exploiting nanodrug formulation was designed and used in the clinic [175]. Later, other complex NPs were developed based on size difference, hypoxia-, acid-, redox-, and protease-responsiveness, among other. Complex NPs were also made based on several tumor features, forming an advanced generation of multifunctional and multi-responsive NPs capable of increasing specificity and efficiency with reduced toxicity. However, these tumor features have not been very effective for conventional chemotherapy, as drugs that are designed to inhibit cell proliferation can lead tumor cells to enter a state of ‘tumor dormancy’ and become resistant to these drugs [174]. Even drug resistance can still occur in the absence of a dormant state. The failure of activable prodrugs to reach the clinic also implies that tumor features look more like a mirage than a miracle for NP delivery. This notion can be supported by recents meta-analysis reports stating that only 0.7 and 0.0014% of systematically injected nanoparticles enter the tumor tissue and tumor cells, respectively, which is a very disappointing result [176]. Another study showed that the amount of NPs reaching the targeted site can be as low as 0.0001% [17]. Additional, studies have demonstrated that the active route for NP extraversion is indeed through active transport rather than EPR [175]. Thus, after 30 years of research, the significance of EPR as the most important feature of tumors to be exploited for nanodrug delivery remains weak. This could be said as well for other features of tumors, such as acidity and hypoxia, due to tumor inter- and intratumor heterogeneity, which fail equal drug distribution in tumor tissue; thus, NPs made based on tumor features may not deliver the optimum results as predicted, as long as tumor dynamics are not fully understood and applied in NP formulation.

**Fig. 17:**
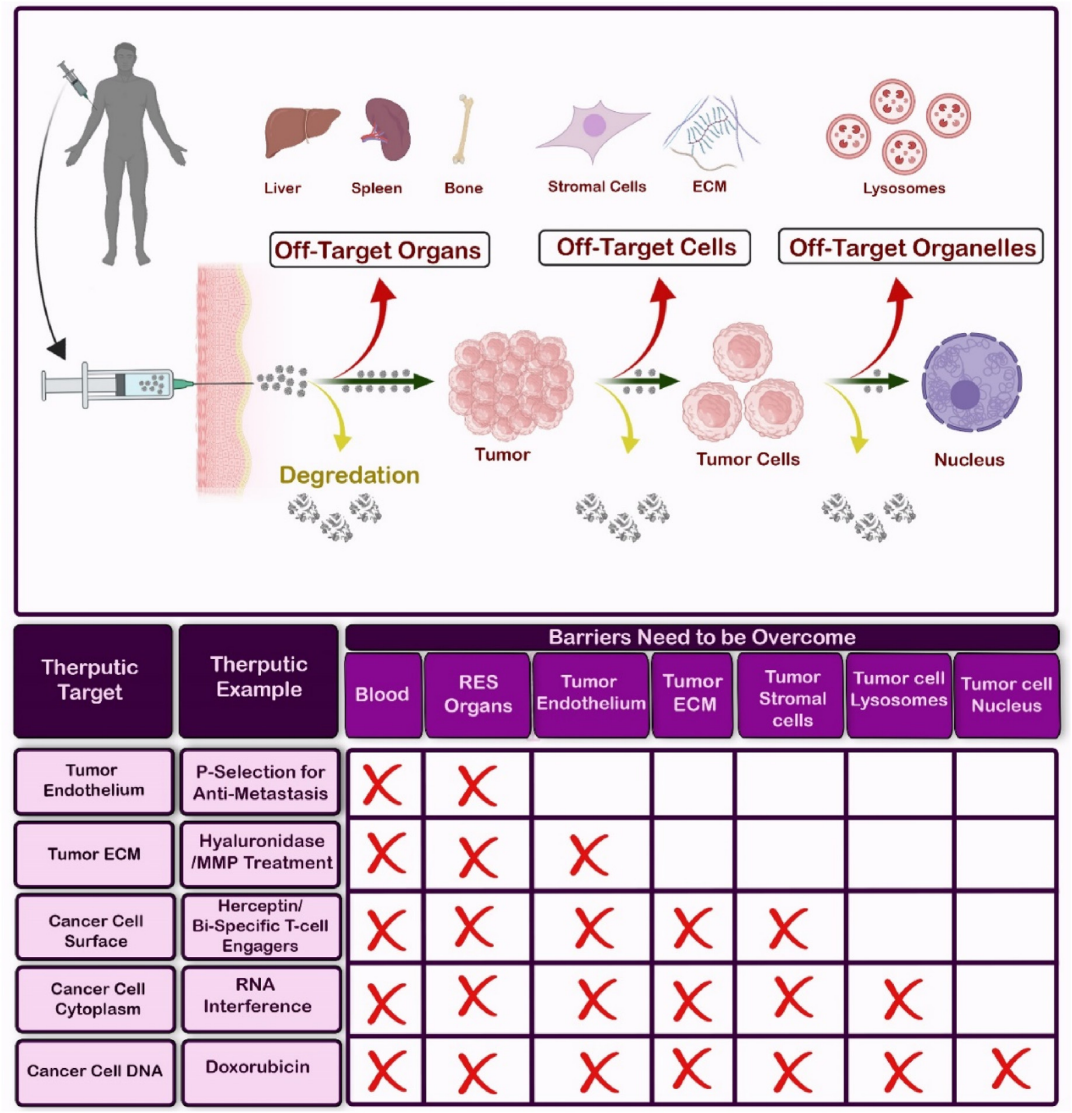
Schematic of All of the biological barriers to targeted delivery of NPs. The body eliminates most of the NPs before it reaches the targeted region.

The number of biological barriers and how well NPs interact with them determine delivery efficiency [177] (Fig. 17). In tumor cell targeting, the number of NCs decreases when they pass from each biological barrier. Conceptually, it makes sense that there is less accumulation as NCs move from the entire tumor to the nucleus of the cancer cell. In most cases, barriers in nano/bio interactions prevent NPs from reaching the desired site. The number of NPs on the way to the delivery site is reduced at each biological barrier. Therefore, understanding the interactions between biological environement and NPs at each level can help to design efficient NCs. Identifying the optimal NP physicochemical properties for delivery to a specific target site is a major challenge. The ideal NP design avoids off-target interactions and favors on-target interactions. In order to achieve this, researchers need to collect and process nano/bio interaction data. In the last decade, a significant number of studies have aimed to elucidate the relationship between the physicochemical properties of engineered NPs and their interaction with biological systems. Nevertheless, there are a complex set of interactions that cannot be defined with a single parameter. For example, the results of in vitro studies can be used for the understanding of nano/bio interactions at the cellular and subcellular levels [17]. In addition, data from in vivo studies are critical to differenciate the role of different organs or systems in the targeted delivery process. NP interactions at the tumor site are also important to understand. Studies have shown that the physiology of the tissue at the tumor site will be different from that of healthy tissue, which can affect NP delivery to the target organ and cells. Therefore, collecting nano/bio-interaction data is an important key step in the optimal design of NPs [178]. The abundance of data will likely require computational analysis to identify how the complex relationships between NPs and biology allow for the identification of the optimal design of NCs. Computational techniques such as linear regression models, machine learning, support vector machines, can be used to process nano/bio interaction data. In addition, identifying the best NP design can be aided by experimentally examining how molecules, cells and tissues interact with nanoparticles of different designs [[179], [180], [181]].

Technology advances, single-cell approaches to cancer research, and the convergence of fields like nanotechnology in biomedicine have made personalized medicine vital for cancer treatment. Personalized therapy may aid cancer patients by reducing treatment issues caused by various cancer cells [182]. Heterogeneity makes cancer treatment harder. Indeed drug-delivery barriers can vary from patient to patient and provide different results. Drug delivery focuses on increasing local NP accumulation, while tumor heterogeneity makes tailored therapy even more critical. Due to the wide range of heterogeneity (evolution and ecology) in diverse cancers and its effect on nanoparticle pharmacodynamics and pharmacokinetics, personalized medicine predicts that the therapeutic outcome will be different for a specific kind and NP formulation with no guarantees. In fact, before NP design and formulation, must be examined the tumor heterogeneity for the best results [28,178,183].

**Fig. 18:**
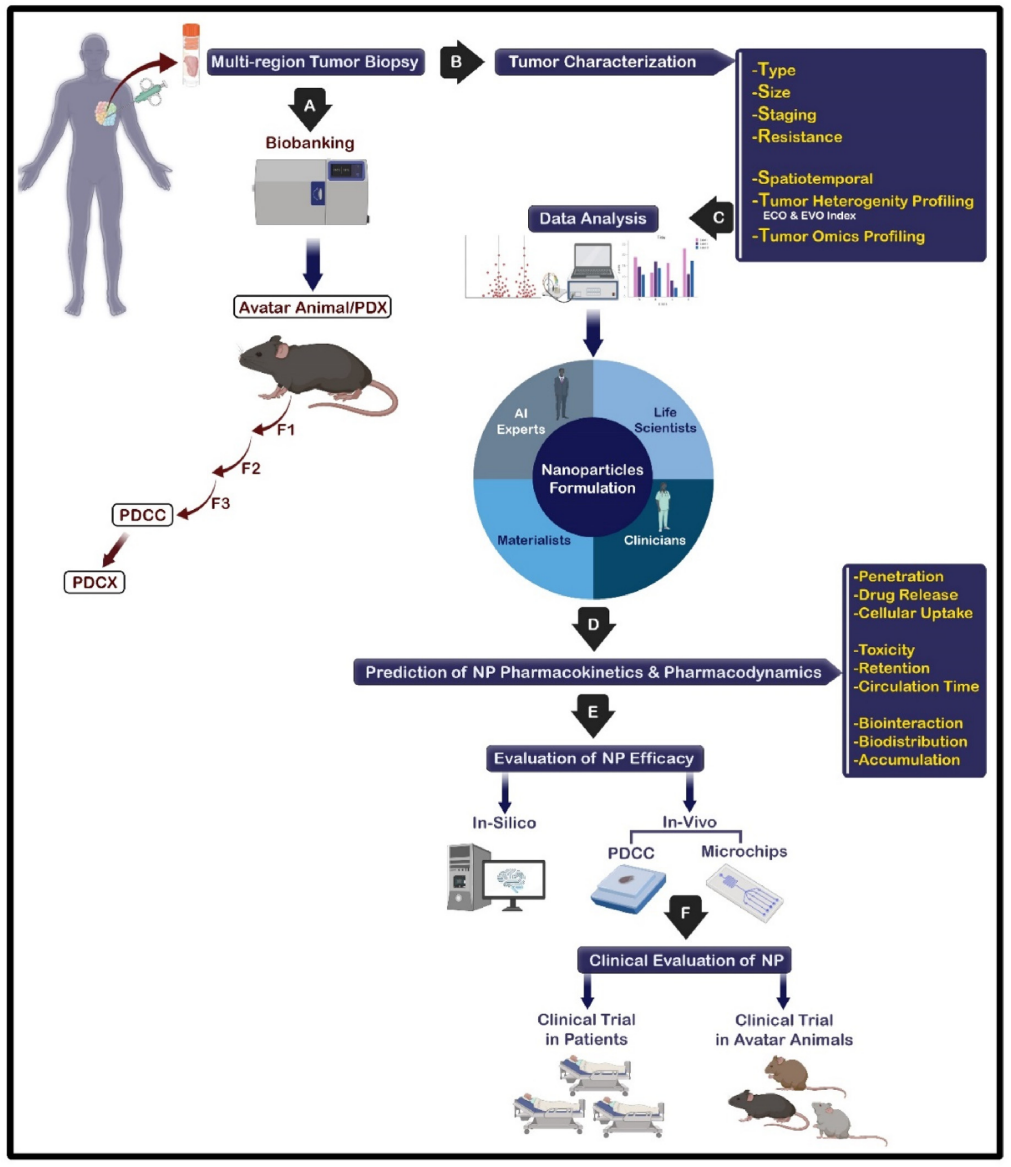
Customized medication NPs. (A) Samples of the solid tumor are biobank. Slides of solid tumors (avatar animal/patient-derived xerographs, or PDX) are explanted into immunocompromised mice. After three passes, patient-derived cell line cultures (PDCCs) are injected into mice to make drug-testing patient-derived cell xerographs (PDCXs). (B) Single-cell techniques and spatiotemporal heterogeneity imaging are utilized in parallel to assess tumor specimen Eco/Evo heterogeneity. (C) Additional field professionals analyze data. (D) Additional NPs tailoring based on tumor characterization and NP pharmacodynamic and pharmacokinetic predictions based on tumor tissue features. (E) Nanoparticles are tested in vitro and silico, and if early results are positive (F), clinical testing is done.

Tumor heterogeneity may make avatar animals unsuitable for co-clinical trial models. Materialists, life scientists, physicians, and artificial intelligence experts must collaborate to build a shared lexicon for personalized medicine-based nano-drug delivery for cancer treatment. Artificial intelligence, single-cell, and microfluidic techniques can accelerate the process. Step-by-step formulations maximize NP localization and adaptation to heterogeneity [[184], [185], [186]], regulating tumor heterogeneity plasticity and clonal selection to prevent recurrence and metastasis (Fig. 18).

For decades, scientists have researched nanoparticle design for cancer treatment delivery. Due to these issues, few have reached the bedside. Despite their benefits, nanostructures must overcome several biological barriers to reach the target area, which may exclude their use for tumor-targeted medicine delivery. In addition to personalized medicine, we can use an advanced strategy to design nanostructures in order to overcome the cascade of biological barriers. To address this, Gisbert-Garzarán et al. designed and developed stimuli-responsive mesoporous silica nanostructures (MSNs) that release drug content slowly, showing enhanced tumor accumulation, cancer cell targeting, and endosomal escape capabilities. For cell targeting and endolysosomal escape, MSN surfaces were modified by biotin and histidine, respectively. Tumoral model studies have shown excellent cytotoxic profiles and remarkable cellular uptake as well as endosomal escape [187]. Wendong et al. designed block copolymer prodrugs (BCPs) with high drug loading, sequential pH, and reduction-responsive properties to overcome biological barriers. This work produced a thiolactone-functional medicine and added functional moieties with amine and pyridine disulfide groups to react. Disulfide linkages and imidazole groups, pH and GSH-sensitive, helped cells internalize and release the polymer prodrug. PTX-based BCPs performed remarkably well in blood flow, tumor accumulation, penetration, and cellular internalization [188]. Passive diffusion-based methods overcome biological barriers, yet medicine administration is still ineffective. Artificial nanomotors can actively search, navigate, and represent targets. Drug delivery using a single motor propulsion mode to pass biological barriers is appealing. Xiang Zhou et al. constructed cascaded Janus calcium carbonate-based micromotors (JCPMs, responsive to light/gas) to overcome multistage biological obstacles for active drug delivery. NIR light and CO_2_ velocity can activate these micromotors. The light/gas double properties of JCPMs enabled multistage and self-adapted biological barrier-breaking during targeted medicine administration [189]. Using nanocarriers to deliver therapeutic and imaging chemicals, researchers are developing targeted techniques and stimulating stimulus-responsive designs to overcome physiological hurdles such as tumor heterogeneity and the complicated tumor microenvironment (some of them are reviewed in) [190,191]. Another strategy is the use of multifunctional NCs to multi-target and combine therapeutic agents, overcome biological barriers, and increase pharmacological properties compared to free therapeutic agents (Reviewed in) [192].

## Declaration of competing interest

The authors declare that they have no known competing financial interests or personal relationships that could have appeared to influence the work reported in this paper.

## Data Availability

Data will be made available on request.
